# Phytochemical Screening, Antioxidant, and Enzyme Inhibitory Properties of Three *Prangos* Species (*P. heyniae*, *P. meliocarpoides* var. *meliocarpoides*, and *P. uechtritzii)* Depicted by Comprehensive LC-MS and Multivariate Data Analysis

**DOI:** 10.3390/antiox11091712

**Published:** 2022-08-30

**Authors:** Stefano Dall’Acqua, Stefania Sut, Gokhan Zengin, Gregorio Peron, Fevzi Elbasan, Evren Yildiztugay, Nabeelah Bibi Sadeer, Mohamad Fawzi Mahomoodally

**Affiliations:** 1 Department of Pharmaceutical and Pharmacological Sciences, University of Padova, Via Marzolo 5, 35131 Padova, Italy; 2 Department of Biology, Science Faculty, Selcuk University, Konya 42300, Turkey; 3 Department of Molecular and Translational Medicine (DMMT), University of Brescia, Viale Europa 11, 25123 Brescia, Italy; 4 Department of Biotechnology, Science Faculty, Selcuk University, Konya 42300, Turkey; 5 Department of Soil Science and Plant Nutrition, Selcuk University, Konya 42300, Turkey; 6 Department of Health Sciences, Faculty of Medicine and Health Sciences, University of Mauritius, Réduit 80837, Mauritius; 7 Center for Transdisciplinary Research, Department of Pharmacology, Saveetha Dental College, Saveetha Institute of Medical and Technical Science, Chennai 600077, India; 8 Centre of Excellence for Pharmaceutical Sciences (Pharmacen), North West University, Potchefstroom 2520, South Africa

**Keywords:** *Prangos*, antioxidants, enzyme inhibitors, phytochemicals, medicinal plants

## Abstract

The aim of the present study was to identify/quantify bioactive compounds and determine the antioxidant activity and enzyme inhibitory effects of various solvent extracts (n-hexane, ethyl acetate, methanol, and water) of *Prangos heyniae* H. Duman and M.F. Watson, *Prangos meliocarpoides var. meliocarpoides*, and *Prangos uechtritzii* Boiss. and Hausskn. This is the first time such a report has been designed to validate the phytochemical composition and bioactivity (especially enzyme inhibitory properties) of these plants. A combined approach of liquid chromatography (LC) with mass spectrometry (HR-MS and MS^n^) allowed to identify that *P. heyniae* contains condensed tannins; *P. meliocarpoides* is rich in hydrolysable tannins; and P. *uechtritzii* possesses coumarins, flavonoids, and hydroxycinnamic acids. Different extracts were tested for antioxidant activities using a battery of assays, such as 2,2-diphenyl-1-picrylhydrazyl (DPPH), 2,2-azino-bis (3-ethylbenzothiazoline-6-sulfonic acid) (ABTS), ferric reducing antioxidant power (FRAP), cupric reducing antioxidant capacity (CUPRAC), total antioxidant capacity (TAC) (phosphomolybdenum), and metal chelating. Enzyme inhibitory effects were investigated using acetylcholinesterase (AChE), butyrylcholinesterase (BChE), tyrosinase, α-amylase, and α-glucosidase as target enzymes. The obtained results depended on the extraction solvents used for each *Prangos* species. The methanol extract of *P. meliocarpoides* var. *meliocarpoides* exhibited significant radical scavenging activity (DPPH: 52.27 mg Trolox equivalent (TE)/g; ABTS: 92.84 mg TE/g), the most potent-reducing potential (CUPRAC: 154.04 mg TE/g; FRAP: 104.34 mg TE/g), and high TAC (2.52 mmol TE/g). Moreover, the strongest BChE (7.97 mg galantamine equivalent/g), α-amylase (0.46 mmol acarbose equivalent/g), and tyrosinase (81.15 mg kojic acid equivalent/g) inhibitory effects were observed for the hexane extract of *P. meliocarpoides* var. *meliocarpoides*. Correlation analysis showed a significant positive correlation between hydrolysable tannins and antioxidant activities. The same trend was also observed between the same class of compounds and the inhibitory effects on enzymatic activities. These results suggest a principal role of hydrolysable tannins in the observed bioactivities of *Prangos*. Our results suggested that the tested *Prangos* species could be valuable as sources of natural agents in the development of health-promoting applications.

## 1. Introduction

Plants are still significant sources of bioactive constituents, and many of the known secondary metabolites still need a lot of research to assess their potential usefulness as therapeutic agents. In the recent time, the development of new therapeutic agents has considered the so-called drug-repositioning and, in this regard, natural products show great potential because they have been used for various medical purposes for thousands of years [1,2]. Furthermore, many natural compounds have been investigated only for limited bioactivities, and they still can be used to assess new potential effects. Humans have used medicinal herbs empirically, with no rational knowledge of their pharmacological effects or active ingredients, but just learning from the experience. Plant-based rational drug discovery began in the early 19th century [3], trying to combine the chemical composition with bioactivity. Despite the importance of biotechnological drugs and monoclonal antibodies, medicinal plants still offer a unique opportunity to discover novel drugs thanks to their exceptional chemical diversity [4].

Plants are a rich source of chemicals, including flavonoids, anthocyanins, carotenoids, catechins, cinnamic acid derivatives, chalcones, stilbenes, and tocopherols, which can promote health through antioxidant action [5]. Phytochemicals can operate as multiple target molecules and can be valuable sources of health-promoting agents, particularly knowing that the pathophysiology of many illnesses is multifactorial and not caused by a single factor [6]. To ensure product safety, particular attention should be paid to pharmaceutical formulation, extraction and manufacture, and mode of action [7]. In this respect, medicinal plants must be investigated to identify possible antioxidants or enzyme inhibitors for use as nutraceuticals, functional foods, or medications.

The genus *Prangos* includes 45 species worldwide and the Irano-Turanian region is the gene center of the genus [8]. The genus is represented by 19 species in Turkey and most of them are distributed in the Central and East Anatolia Region [9]. In the literature, several studies have been performed on the members of the genus *Prangos*. For example, crude extracts or essential oil of *P. ferulacea* (L.) Lindl. are commonly investigated in terms of its volatile and non-volatile components, cytotoxic activity; antioxidant, antimicrobial, hypoglycemic, acetylcholinesterase, and analgesic activities [10,11,12]; wound healing property [13]; and antiviral activity [14]. Interestingly, a randomized controlled trial was even conducted to determine the effect of a vaginal cream containing *P. ferulacea* on accelerating the recovery of bacterial vaginosis. The results showed that the cream displayed a positive effect on patients with this type of inflammation [15]. A novel coumarin (yuganin A) identified in the roots of *P. pabularia* Lindl. exhibited potent effects on the proliferation of B16 melanoma cells [16]. *P. haussknechtii* Bioss containing coumarins 1 and 2, monoterpenoids, and amino acid derivatives inhibited lipid peroxidation with IC_50_ values between 43 and 114 μM, and reduced MTT to formazan blue between 48 and 128 μM [17]. Tan, et al. [18] assessed the antibacterial activity of the pyrenylated coumarin 4′-senecioiloxyosthol, identified in the roots of *P. hulusii* Senol, Yildirim, and Secmen. The results showed that the coumarin exhibited antimicrobial activity against *Bacillus subtilis* at a minimum inhibitory concentration of 5 µg/mL. The aim of this study is to assess the bioactive compounds and biological activities of three underexplored *Prangos* species, namely *P. heyniae* H.Duman and M.F.Watson, *P. meliocarpoides* var. *meliocarpoides*, and *P. uechtritzii* Boiss. and Hausskn. In the literature, the *Prangos* species have been studied for several biological abilities. For example, Ahmed et al. [19] investigated the total phenolic and DPPH radical scavenging abilities of the methanol and water extracts of the three *Prangos* species, and the water extract of *P. heyniae* was the richest in terms of total phenolic level, with 127.33 mg GAE/g. In addition, the extract was the most active one in DPPH radical scavenging ability with the lowest IC_50_ values (20.96 µg/mL). In a recent study performed by Albayrak et al. [20], two new coumarin glycosides (7-methoxy isoarnottinin 4′-*O*-β-d-glucopyranoside and 7-methoxy isoarnottinin 4′-*O*-rutinoside) were isolated from the roots of *P. heyniae* collected from Turkey. In addition to the studies on the extract of *P. heyniae*, several studies showed chemical characterization and some biological activities of the essential oil of the species [21,22]. In an earlier study conducted by Oke et al. [23], the radical scavenging and chemical composition of *P. meliocarpoides* fruit extracts were determined and the water extract was found to be the most active one. Moreover, chlorogenic and rutin were determined to be the main components in their study. The fruit extract had a higher radical scavenging ability as compared with the root and aerial parts in another study [19]. Among the targeted *Prangos* species, *P. uechtritzii* has been the most studied one and several studies reported its essential oil composition as well as biological activities [24,25,26]. A recent study by Sevin et al. [27] examined the erectile function of root extract of *P. uechtritzii* as well as *P. heyniae*, and the authors reported that oxypeucedanin was the most active coumarin. In our earlier paper [28], the chemical characterization and biological activities of the essential oils of three *Prangos* species were reported. Although many studies have examined the phytochemical composition and bioactivity of different *Prangos* species, the three species examined here still need to be evaluated and confirmed by scientists. To the best of our knowledge, this study is the first to report the phytochemical composition, antioxidant effects, and inhibitory activity against α-amylase, α-glucosidase, acetylcholinesterase, butyrylcholinesterase, and tyrosinase enzymes of secondary metabolites extracted from the same species. We believe that the results presented here could fill in the research gap and subsequently open new research avenues, particularly in the development of therapeutic bioproducts.

## 2. Materials and Methods

### 2.1. Plant Material and Extraction

The aerial parts of the *Prangos* species were collected during Summer 2020 from Central Anatolia Region of Turkey. The information of the collection area was reported in our earlier paper [28]. Voucher specimens were deposited at the herbarium of Selcuk University, Science Faculty (Voucher numbers: EY-3039, EY-2998, and EY-3023 for *P. heyniae*, *P. meliocarpoides var. meliocarpoides*, and *P. uechtritzii*, respectively). The plant samples were dried in shade conditions at room temperature for about one week. Then, the powdering procedure was performed using a mill, and the samples were stored in the dark.

Extracts were prepared using n-hexane, ethyl acetate, methanol, and water. Overnight, the plant material (10 g) was macerated at room temperature with 200 mL of solvents (hexane, ethyl acetate, and methanol, individually). Finally, solvents were evaporated from the mixtures. Plant materials (10 g) were extracted in 200 mL of boiling water for 15 min before being filtered. Water extracts were lyophilized. All extracts were stored at 4 °C until further analysis.

### 2.2. Total Phenolic and Flavonoid Contents

The total phenolic and flavonoid contents were determined using the Folin–Ciocalteu and AlC_l_3 tests, respectively [29]. The results were presented as gallic acid equivalents (mg GAEs/g dry extract) and rutin equivalents (mg REs/g dry extract) for the assays. The experimental details are given in the supplemental section.

### 2.3. LC-DAD-MS^n^

Extracts were dissolved in appropriate solvent: the more lipophilic extracts were dissolved in DMSO, methanol extracts in methanol, and water extracts were dissolved in water/DMSO mixtures. Samples were prepared weighting 10 mg (±0.1 mg) of dried material and dissolved in 10 mL of solvent using an ultrasound bath (WWR, Ultrasonic bath 45 Hz, 60 W, WWR Milano, Italy) for 15 min [30]. Then, the volume was adjusted at 20 mL and solutions were centrifuged at 13,000 rpm prior to being transferred to glass vials for analyses [30].

LC-DAD-MS^n^ analyses were performed using an Agilent 1260 chromatograph equipped with autosampler, column oven, and diode array detector (DAD), all from the 1260 series. At the end of the chromatographic column, a “T” connector was fixed splitting the flow into two identical volumes: one was linked to DAD, while the other was connected to the electro spray (ESI) ion source of a Varian 500 Ion Trap mass spectrometer (MS). The spectrometer operated in both negative and positive ion modes. Data were acquired in the 100–2000 *m*/*z* range using the turbo data depending scanning (tdds) function, which allows the recording of the fragmentation of the most abundant ionic species. MS parameters were as follows: needle voltage: 4800 V, capillary voltage: 80 V, drying gas pressure: 25 psi, nebulizer pressure: 40 psi, drying gas temperature: 280 °C, and nebulizer temperature: 270 °C. For the identification of compounds, MS^n^ spectra were acquired and compared with the literature and available online databases (Human Metabolome Database (HMDB) and Food Metabolome Database (FOodB)). Furthermore, MS data were compared with those of reference compounds available in the laboratory or acquired by commercial sources. Quantification of compounds was obtained using selected reference standards for each class of identified constituents. The calibration curves were as follows: imperatorin (detected in MS, positive ion mode, and DAD at 320 nm), reference solutions from 90 to 1.5 µg/mL, y = 35x − 0.320; rutin (detected in MS, negative ion mode, and in DAD at 350 nm), reference solutions from 100 to 1.0 µg/mL, y = 72x − 1.20; ellagic acid (detected in MS, negative ion mode, and in DAD at 254 nm), reference solutions from 100 to 1.0 µg/mL, y = 37.5x − 0.88; and epigallocatechin gallate (detected in MS, negative ion mode, and in DAD at 280 nm), reference solutions from 120 to 1.0 µg/mL, y = 83x − 0.992.

Accurate *m/z* values of identified metabolites were obtained by UPLC-QToF analysis of the same *Prangos* extracts. The system used for the analysis was an Acquity UPLC (Waters) coupled with a QToF MS model Xevo G2 (Waters). Agilent Zorbax Eclipse Plus C18 (2.1 × 50 mm, 1.8 μm) was used as the stationary phase, which was kept at a constant temperature of 40 °C. As the mobile phase, a mixture of water with 1% formic acid (A) and acetonitrile (B) was used. The elution gradient started from 0 to 1 min, 98% A; then at 11 min, 15% A; at 16 min, 0% A; and kept isocratic until 20 min. Then, at 21 min, 98% A and 24 min, 98% A. The flow rate was 300 µL/min, and the injection volume was 2 μL. MS data were acquired in both ESI(+) and ESI(−) in the mass range of 50–2000 Da. The sampling cone voltage was adjusted at 40 V and the source offset was 80 V. The capillary voltage was set to 3.5 KV. Nitrogen was used as nebulizer gas at a flow rate of 800 L/h. The desolvation temperature was 450 °C. Mass accuracy and reproducibility were maintained by infusing lock mass (leucine–enkephalin, [M + H]^+^ = 556.2771 *m/z*, and [M − H]^−^ = 554.2620 *m/z*) through Lockspray at a flow rate of 20 μL/min. The *m/z* value of all acquired spectra was automatically corrected during acquisition based on lock mass. MS^e^ experiment was simultaneously performed to collect structural information, setting the collision energy to 30 V.

### 2.4. Antioxidant Assays

Antioxidant assays were performed using methods that have been previously reported [31,32]. The antioxidant potential was calculated as follows: mg Trolox equivalents (TE)/g extract in the 2,2-diphenyl-1-picrylhydrazyl (DPPH) and 2,2′-azino-bis(3-ethylbenzothiazoline-6-sulfonic acid) (ABTS) radical scavenging tests; cupric-reducing antioxidant capacity (CUPRAC) and ferric-reducing antioxidant power (FRAP); and mmol TE/g extract in the (MCA). The experimental details are given in the supplemental section. 

### 2.5. Enzyme Inhibitory Assays

The enzyme inhibition experiments were performed based on previously described procedures [31,32]. Amylase and glucosidase inhibition was expressed as mmol acarbose equivalents (ACAEs)/g extract, while acetylcholinesterase (AChE) and butyrylcholinesterase (BChE) inhibition was expressed as mg galanthamine equivalents (GALAEs)/g extract. Tyrosinase inhibition was expressed as mg kojic acid equivalents (KAEs)/g extract. The experimental details are given in the supplemental section. 

### 2.6. Statistical Analysis

This study used ANOVA (Tukey’s test) in order to determine whether there were any differences in the extract levels between the three samples. xlStat was used to carry out the statistical analysis. *p*-values < 0.05 were considered as statistically significant. Multivariate statistical analyses were performed on chemical and biological data to uncover correlations between chemical constituents of *Prangos* extracts and their bioactivities, i.e., to describe possible activity biomarkers. Predictive OPLS models were developed using SIMCA (Umetrics, Sweden), and they were validated performing a permutation test (*n* = 200 permutations). Pearson’s rank correlation test was performed using the open-source MetaboAnalyst platform (https://www.metaboanalyst.ca/, accessed on 14 July 2022). Prior to being analyzed, data were pre-processed as follows: variables with more than 80% missing values were removed, and the remaining missing values were imputed using the KNN algorithm; finally, they were normalized by log-transformation and Pareto scaling. 

## 3. Results and Discussion

### 3.1. Characterization of Bioactive Secondary Metabolites

The choice of the most suitable extraction solvent is an important step in order to define the quality and yield of extraction of these compounds [33]. Solvents used to extract bioactive chemicals from plants are chosen based on the polarity of the solute of interest, because a solute with comparable polarity to the solvent will be adequately dissolved according to the rule of similarity and intermiscibility (like dissolves like) [34,35]. In this study, the polyphenolic compounds such as phenolics (TPCs) and flavonoids (TFCs) of *P. heyniae*, *P. meliocarpoides* var. *meliocarpoides*, and *P. uechtritzii* extracted with four solvents of different polarities (water > methanol > ethyl acetate > hexane) were quantified. The results are presented in Table 1. 

Overall, we observed a decline in TPC and TFC in the different plant species studied in the following order: *P. meliocarpoides* var. *meliocarpoides* (TPC: 44.28 mg GAE/g, and TFC: 44.66 mg RE/g), *P. heyniae* (TPC: 38.77 mg GAE/g, and 28.75 mg RE/g), and *P. uechtritzii* (TPC: 31.20 mg GAE/g, and TFC: 28.22 mg RE/g). The most polar solvents, namely water and methanol, were successful in extracting phenolics and flavonoids. As such, it can be said that the compounds extracted were mostly polar. On the other hand, hexane extracted the least number of compounds. A number of publications have reported methanol and water as the most efficient solvents in obtaining a high yield of extracts, as well as in extracting polar bioactive compounds such as phenolics and flavonoids [36,37].

The bioactive compounds in *Prangos* species were investigated by combining positive and negative ion mode LC-MS (Table 2). The analysis revealed that the extraction of *Prangos* species yielded extracts containing different classes of secondary metabolites, which we grouped as condensed tannins, hydrolysable tannins, coumarins, flavonoids hydroxycinnamic acid derivatives, and “other constituents”. Schematic representations of the relative abundance of the main constituents are reported in the form of pie-charts in Supplementary Materials Figures S1–S3. The structures of the identified compounds were deduced owing to the HR-MS data as well as by MSn. Epigallocatechin gallate derivatives are characterized by the loss of one or two gallic acid moieties (*m/z* 152), leading in this latter case to ion *m/z* 305, corresponding to the epigallocatechin moiety [38,39] (Table 1). Other condensed tannins in *P. heyniae* were prodelphynidine gallate derivatives, compounds in fact present the molecular formula of C_37_H_30_O_18_, which generate fragment because of −152 Da ascribable to gallic acid moiety. Further fragmentations of the species generated at *m/z* 611 are ascribable to prodelphynidin dimer gallate. Four different isomeric derivatives are present in *P. heyniae.* Two further peaks presenting similar fragmentation and showing the loss of two gallic acid moieties have also been detected and annotated as di-galloyl prodelphynidin dimers [40,41,42]. Epigallocatechin gallate and methyl ellagic acid were also detected and their presence was confirmed with an injection of authentic standards. An unusual derivative presenting molecular formula of C_14_H_12_O_11_ was tentatively assigned to Chebulic acid due to the loss of water and CO_2_,leading to the fragment at *m/z* 293 that further presented loss of further CO_2_ moieties leading to the species at *m/z* 249, 205, and 163; the mass spectra are reported in Supplementary Figure S4. A second peak presenting the same molecular ion and fragmentation pathway was observed and indicated as a chebulic acid isomer (Supplementary Figure S5). These compounds have been identified in the fruits of *Terminalia chebula* and LC methods have been proposed for their identification [43]. A series of condensed tannins formed by units of epigallo/gallo catechin with or without gallic acid esterification have been also identified [39,44,45]. Furthermore, the peak presenting molecular formula of C_44_H_36_O_22_ was tentatively assigned to the Assamicain chalcan flavan dimer previously identified in *Camelia sinensis* [46]. Several hydrolysable tannins have been identified specifically in *P. meliocarpoides* var. *meliocarpoides* and most of the compounds were ester of gallic acid with glucose, quinic acid; moreover, different derivatives formed by mixed esters of gallic, synaptic, and shikimic acid have been detected, and identification was based on the obtained MS measurements and predicted MS fragmentation from database and software, as well as by comparison with the literature [44,47,48,49,50]. Some coumarin derivatives were identified, especially in the *P. meliocarpoides* extracts obtained with methanol or water. Coumarins have been reported in *Prangos* species [8,51], where the identity of compounds was confirmed with the injection of reference purified substances. A series of glycosidic flavonoids, mainly quercetin, myricetin, and isorhamnetin derivatives, were also identified [52,53] and quantified, and they mostly were present in *P. uechtritzii*.

As clearly shown in Supplementary Figures S1–S3, composition is different considering the various species and extraction solvents. Chromatograms are also given in Supplementary Figures S5 and S6. *P. heyniae* extracts are characterized by large abundance of condensed tannins (except for the hexane extract, which is rich in 6-hydroxy-3-oxo-alpha ionol), while *P. meliocarpoides* mostly contains hydrolysable tannins. More complex is the situation of *P. uechtritzii*, which presents coumarins in the hexane extract, other constituents in the ethyl acetate extract, and flavonoids in methanol and water extracts. 

Thus, the three *Prangos* species differ in composition and the solvent used for the extraction can influence the composition of the obtained products. Positive and negative ion mode LC-MS revealed that the most abundant compounds in the most lipophilic extracts (hexane and ethyl acetate) of *P. heyniae* are 6-hydroxy-3-oxo-alpha-ionol and the p-hydroxy-benzoic acid rhamnosyl ester. The extraction is influenced by the nature of the solvent and, using methanol and water, other derivatives have been extracted from *P. heyniae* in higher yields such as epigallocatechin digallate, and galloyl prodelphynidine resulted in more abundant compounds of the methanol and water extracts. The *P. heyniae* samples also contained the coumarins heraclenin, 8-methoxy coumarin, and marmesin. Considering *P. meliocarpoides*, all of the different extracts present as the most abundant constituents’ hydrolysable tannins. Nevertheless, chebulic acid (a tricarboxylic acid forming specific ellagitannin derivatives), as well as chebulinic acid (an ellagitannin), were specifically detected only in this species and, to the best of our knowledge, this is the first report of these derivatives in *Prangos*, while previous identification of such compounds occurred in *Terminalia* spp and in *Phyllantus* emblica [54,55]. *P. uechtritzii* is characterized by the presence of flavonoid glycosides and coumarins, while it contains a negligible amount of condensed or hydrolysable tannin.

Some compounds can be considered characteristic for the different species. For example, condensed tannins, mostly derivatives of epigallocatechin gallate and galloprodelphynidine, are found mainly in *P. heyniae.* On the other hand, chebulic acid, i.e., a tetracarboxylic acid, was found only in *P. meliocarpoides*, which is also characterized by the large presence of hydrolysable tannins, which were not detected in the other two species. Considering the coumarins, heraclenin characterized *P. heyniae* while imperatorin and dentatin were specifically found only in *P. meliocarpoides* and *P. uechtritzii*, respectively.

The results revealed that, considering the whole phytochemical profile of extracts obtained from this plant material, methanol and ethyl acetate are the most suitable solvents, despite water and hexane. It is possible that the condensed tannins and epigallocatechin derivatives are not soluble enough, neither in the too nonpolar hexane nor in water. What is notable compared with other *Prangos* species reported in the literature is the limited amount of detected coumarins, as only low amounts of imperatorin were detected. 

A similar behavior was observed for *P. meliocarpoides*. In fact, the LC-MS analysis revealed that more compounds were detected in the ethyl acetate and methanol extracts. The main detected compounds in this second case were tetragalloyl quinic acid and digalloyltheaflavonin. Marmesin and imperatorin were also detected, showing the presence of different coumarins compared with the previous plant species. Furthermore, condensed tannins as punicalagins; the ellagitannin terchebulin; as well as esters of gallic, shikimic, and synapoyl acid with glucose represented the most notable constituents. *P. uechtritzii* extracts were characterized by the presence of a large amount of the coumarins 8-methoxy coumarin and marmesin, as well as a significant amount of epigallocatechin-gallate. Furthermore, this species was the only one showing a pattern of glycosidic flavonoids, myricetin, and quercetin.

### 3.2. Antioxidant Effects

The antioxidant activities of *P. heyniae*, *P. meliocarpoides* var. *meliocarpoides*, and *P. uechtritzii* extracts were evaluated using different assays. In our model, DPPH and ABTS assays were used to assess radical quenching ability, while FRAP and CUPRAC assays results indicated reducing power. The capacity of extracts to chelate ferrous ions was measured by metal chelating assay, while the phosphomolybdenum test was useful to establish the total antioxidant capacity. Each assay presents strengths and limitations, as detailed elsewhere [56]. The results are presented in Table 1 and Table 3. 

Overall, the findings showed that a correlation exist between DPPH, ABTS, FRAP, and CUPRAC assays, except with the metal chelating and phosphomolybdenum assay. Such a correlation was observed in several other studies [56,57,58,59]. The methanolic and aqueous extracts exhibited the highest antioxidant activities in most of the assays, suggesting an important role of the phenolics and flavonoids of the extracts. The polarity-dependent increase in total antioxidant activity and reducing properties indicates that the extraction of strong antioxidant compounds is more favorable in polar solvents [60]. Furthermore, in a previous paper that highly polar solvents, such as methanol, can have a high effectiveness in the extraction of antioxidants [35], supporting our findings. 

Among the tested *Prangos* species, *P. meliocarpoides* var. *meliocarpoides* exhibited the strongest antioxidant activity in DPPH (52.27 mg TE/g), ABTS (92.84 mg TE/g), CUPRAC (154.04 mg TE/g), FRAP (104.34 mg TE/g), and phosphomolybdenum (2.52 mmol TE/g) assays. However, *P. heyniae* displayed the highest metal chelation effects (27.79 mg EDTAE/g) considering metal-chelating properties. Previous studies have also reported a weak correlation between metal-chelating assays and DPPH, ABTS, CUPRAC, and FRAP methods [61,62,63,64]. An explanation of this discrepancy is related to the different chemical reactions involved in the assays. The DPPH, ABTS, CUPRAC, FRAP, and phosphomolybdenum assays are single-electron transfer or hydrogen atom transfer reaction-based assays. On the other hand, the metal-chelating assay involves sequestration of transition metals [56]. This can justify the difference in the results obtained with DPPH, ABTS, CUPRAC, and FRAP methods and metal-chelating tests. 

### 3.3. Enzyme Inhibitory Activities

In the present study, the ability of *P. heyniae*, *P. meliocarpoides* var. *meliocarpoides*, and *P. uechtritzii* extracts to modulate the activity of enzymes related to Alzheimer’s disease (acetylcholinesterase (AChE) and butyrylcholinesterase (BChE)), diabetes type 2 (α-amylase and α-glucosidase), and skin hyperpigmentation (tyrosinase) was investigated, and the results are presented in Table 4.

Although AChE inhibition is regarded as a very promising technique for symptomatic therapy of Alzheimer’s disease, the involvement of BChE in late Alzheimer’s disease has been established [65,66]. Among the prepared extracts, hexane extracts demonstrated the highest anti-AChE and anti-BChE activities for the three *Prangos* species, despite that these extracts yielded the lowest phenolic and flavonoid content. In terms of plants, *P. heyniae* demonstrated the highest inhibitory property against AChE (2.39 mg GALAE/g). However, the three plants exhibited relatively similar activity against BChE, with galantamine equivalent ranging from 7.63 to 7.97 mg GALAE/g. Taken together, the three members of the *Prangos* genus could be considered as valuable sources of AChE and BChE inhibitors. It is important to highlight that, despite that aqueous extracts possessed high levels of phenolic and flavonoid, the extracts were not good inhibitors of AChE and BChE. In an earlier study by Abbas-Mohammadi et al. [67], who tested twenty-five Iranian plants for AChE inhibition, found the n-hexane fraction of *P. ferulacea* to be the most active (75.6%) at 50 µg/mL concentration, and the results are consistent with our presented results, where n-hexane extracts were the most active on AChE. In addition, several furanocoumarins were isolated in their study, which showed more potent AChE inhibitory effects with low IC_50_ values. However, we did not find a good correlation between coumarins and AChE inhibitory properties of the tested extracts. This fact could be explained by the interaction of phytochemicals (antagonistic and so on) in the present study. Thus, we strongly suggested to conduct further studies on the cholinesterase inhibitory properties of isolated compounds from the tested *Prangos* species. In another study by Bahadori [68], the AChE and BChE inhibitory effects of *P. gaubae* extracts (n-hexane, dichloromethane, and methanol) were detected, and the dichloromethane extract was the most active on both enzymes (AChE: 2.62 mg GALAE/g; BChE: 3.51 mg GALAE/g). The AChE inhibitory effects for the methanol extracts of *P. ferulacea* and *P. peucadanifolia* were also reported as 1.47 and 4.09 mg GALAE/g, respectively [69]. 

**Figure 1:**
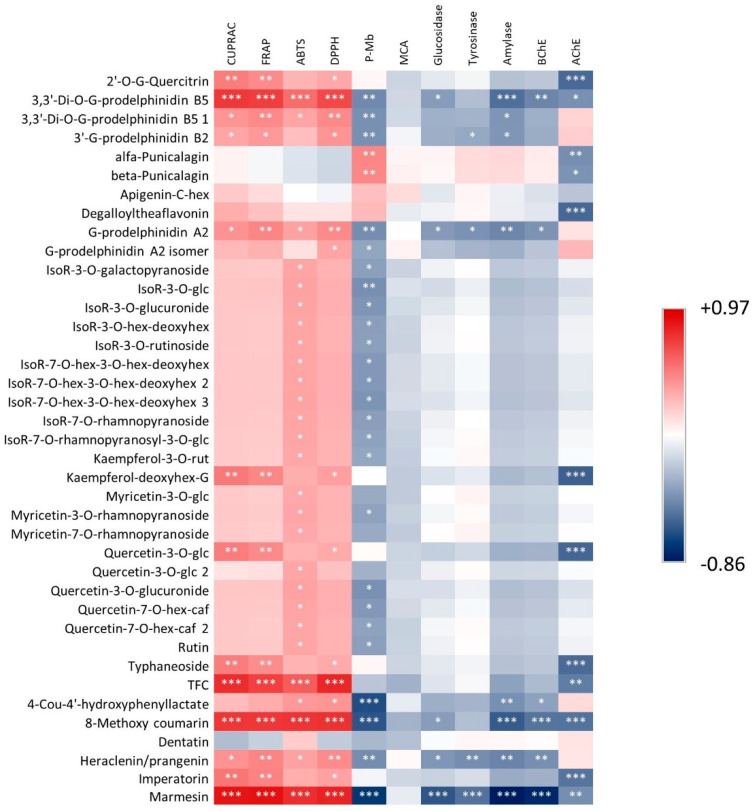
Correlation heatmap showing positive (red colour) and negative (blue colour) correlations between flavonoids and coumarins and measured bioactivities. Color scale is dependent on Pearson’s r value. Significance levels are indicated by asterisks in the plot. *: *p* < 0.05; **: *p* < 0.01; ***: *p* < 0.001.

α-Amylase and α-glucosidase inhibitors delay the breakdown of carbohydrates in the small intestine and, as a consequence, decrease the post-prandial blood glucose level, which is considered as an important treatment strategy to manage blood glucose levels in type 2 diabetic patients [70]. As shown in Table 4, the three *Prangos* species exhibited similar inhibition against α-amylase enzyme (0.40–0.46 mmol ACAE/g). However, the highest anti-glucosidase activity was observed with *P. uechtritzii* (0.78 mmol ACAE/g), followed by *P. meliocarpoides* var. *meliocarpoides* (0.74 mmol ACAE/g) and *P. heyniae* (0.67 mmol ACAE/g). No activity against α-glucosidase was noted with aqueous extracts. Inhibition of α-amylase and α-glucosidase enzymes has been reported to be an intriguing target for the management of type II diabetes because of less side effects when compared with standard therapies [71,72]. Few studies have been reported in the literature on the amylase and glucosidase inhibitory effects of members of the genus *Prangos.* Loizzo et al. [73] tested the amylase and glucosidase inhibitory activity of *P. asperula* extracts (n-hexane, chloroform, and methanol), and the n-hexane extract was the most active for the enzymes, consistent with our findings. However, the best amylase and glucosidase inhibitory activity was detected by dicholoromethane extract (amylase: 0.93 mmol ACAE/g and glucosidase: 20.07 mmol ACAE/g) of *P. gaubae* [68]. The amylase and glucoside inhibitory properties were also reported for *P. ferulacea* (amylase: 0.77 mmol ACAE/g and glucoside: 4.45 mmol ACAE/g) and *P. peucadanifolia* (amylase: 0.83 mmol ACAE/g and glucosidase: 4.97 mmol ACAE/g) [69]. From Figure 1, the observed amylase and glucosidase inhibitory abilities of the tested extracts were also moderately correlated with punicalagins and the compounds were reported as antidiabetic agents in earlier studies [74,75]. 

Tyrosinase is a rate-limiting enzyme that is responsible for the manufacture of melanin, and it is regarded as a critical therapeutic approach for the treatment of skin hyperpigmentation problems [76]. The methanolic extract of *P. meliocarpoides* var. *meliocarpoides* displayed the best tyrosinase inhibitory effect (81.15 mg KAE/g), followed by *P. uechtritzii* (68.03 mg KAE/g) and *P. heyniae* (65.20 mg KAE/g). Aqueous extracts exhibited the lowest inhibition against tyrosinase (Table 4). In a previous study by Zengin et al. [69], the tyrosinase inhibitory properties of the methanol extract of *P. ferulacea* (131.94 mg KAE/g) and *P. peucadanifolia* (128.54 mg KAE/g) were higher than those of the presented study. The tyrosinase inhibitory properties of the extracts of *P. gaubae* varied from 16.85 mg KAE/g (in methanol) to 36.33 mg KAE/g (in n-hexane) [68].

**Figure 2:**
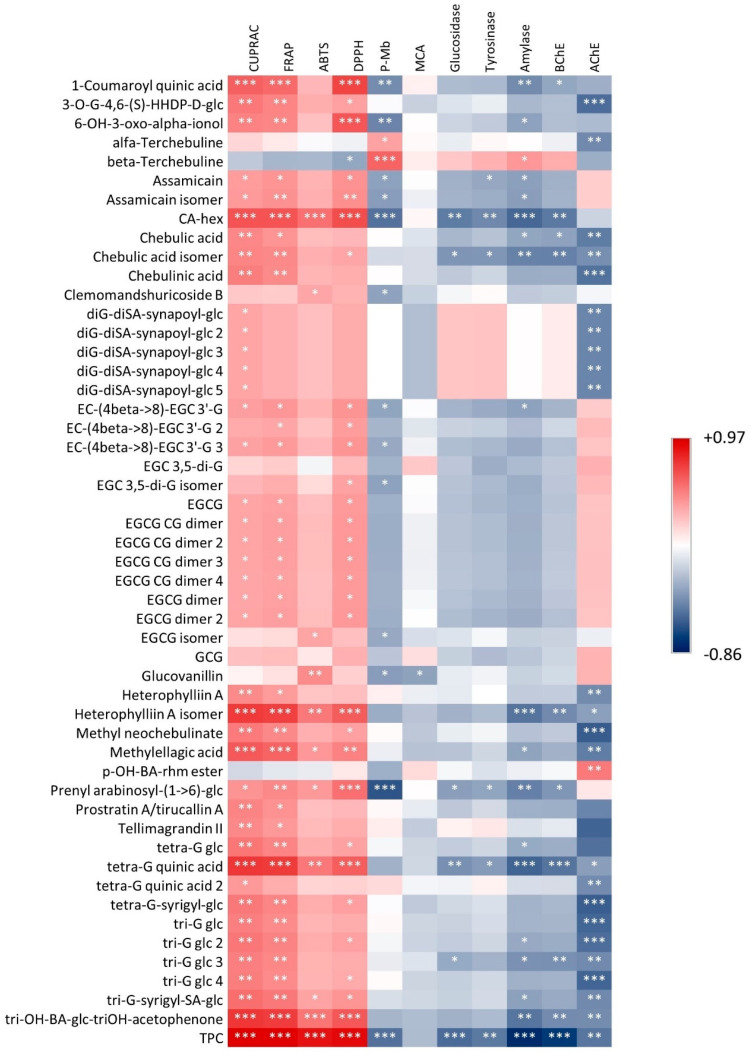
Correlation heatmap showing positive (red colour) and negative (blue colour) correlations between specific polyphenols and measured bioactivities. Color scale is dependent on Pearson’s r value. Significance levels are indicated by asterisks in the plot. *: *p* < 0.05; **: *p* < 0.01; ***: *p* < 0.001.

Heatmaps were generated to establish possible correlations between the chemical composition of extracts and the observed bioactivities. One of these plots considered flavonoids and coumarins, while the second considered all of the other polyphenols (tannins and other constituents). These heatmaps are reported in Figure 1 and Figure 2, respectively. Clearly, the results show a strong positive correlation between antioxidant activities and all of the different tannins, as well as between prodelphynidine and total flavonoid content. Considering the enzymatic activities, epigallocatechin derivatives were moderately correlated with AChE inhibition, suggesting a potential role of these compounds on this specific enzyme. As reported by Jabir et al. [77], different polyphenols have been considered as potential inhibitors of AChE, but their mode of action should be specifically studied, considering not only the possible different interactions with the active site of the enzyme, but also the potential modification to polyphenol structures due to metabolism of the host. Thus, our data suggest a possible role of gallocatechin derivatives as potential inhibitors of AChE, and they can lead to further studies on these natural chemical compounds.

**Figure 3:**
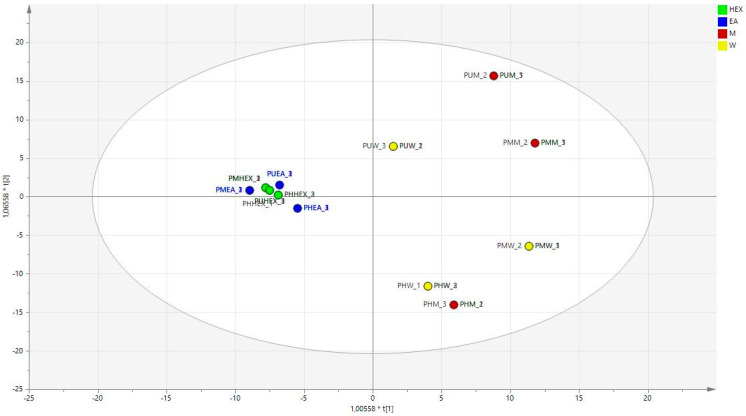
OPLS model considering all of the prepared extracts categorized on the basis of the solvents, HEX: hexane, EA: ethyl acetate, M: methanol, and W: water.

**Figure 4:**
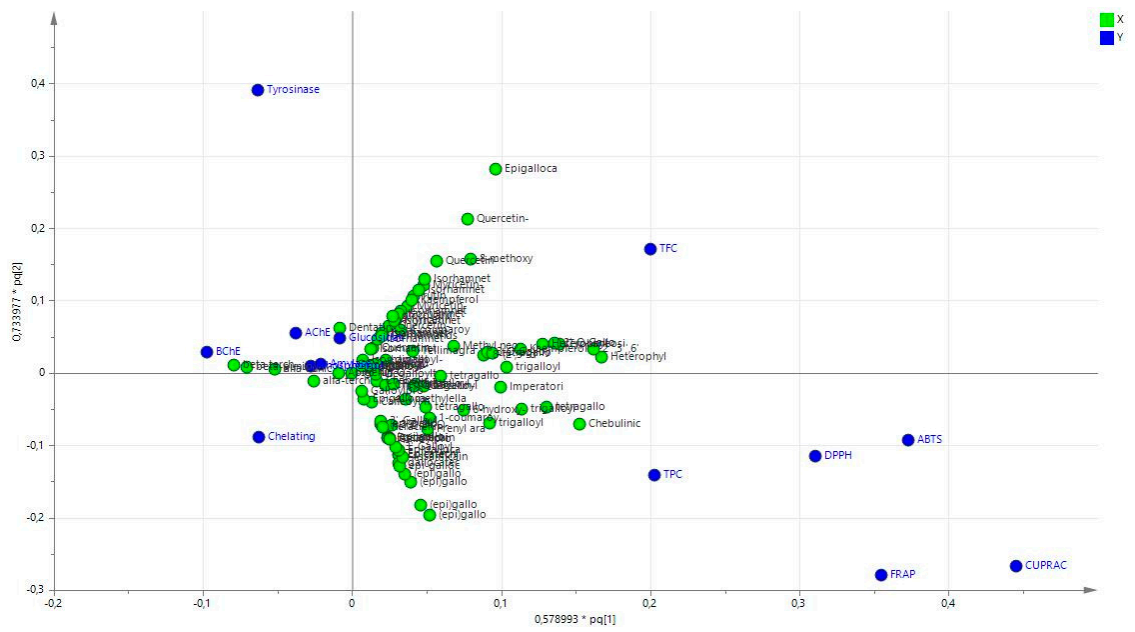
OPLS loading plot considering chemical components and biological properties.

Furthermore, the OPLS model was generated using the data from the chemical analysis and combining the results of the bioassays. Figure 3 shows the score scatter plot, while the loading plot is shown in Figure 4.

**Figure 5:**
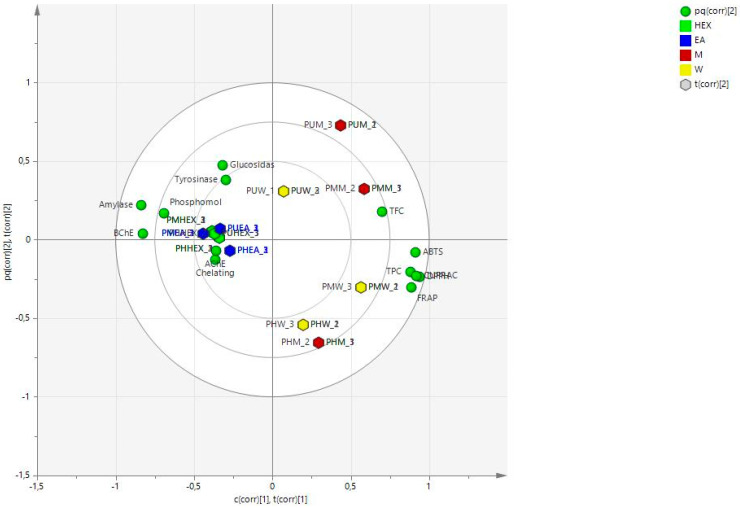
Biplot summarising the results of the assays and the different extracts.

In the score scatter plot (Figure 3), a clear distinction can be observed considering the extracts obtained with the lipophilic solvents, namely hexane and ethyl acetate, and the extract obtained with the more hydrophilic methanol and water, with the first being all in the -x part of the plot and the other at the opposite side. As shown by the loading plot (Figure 4) and in the biplot (Figure 5), the more relevant results related to antioxidant activities are obtained with the methanol and water extracts, while the enzyme inhibitory activities appear to be related mostly to the more lipophilic extracts. This result suggests proceeding with the studies using the water and methanol extracts of *Prangos* species as sources of antioxidant constituents, while using the lipophilic solvents to extract compounds that can be evaluated as inhibitors of cholinesterase, tyrosinase, amylase, or glucosidase. Considering the other assays, we can observe that CUPRAC appears to be related to the hydrophylic extracts, while the metal-chelating assay results were ascribed to lipophilic extracts. Thus, the application of multivariate analysis can be used to select the proper extraction solvent to improve or to search for compounds or mixtures presenting efficient activity on target assays.

## 4. Conclusions

In this study, the different phytochemical composition and biological activities (antioxidant and enzyme inhibitory effects) of three *Prangos* species, namely, *P. heyniae*, *P. meliocarpoides* var. *meliocarpoides*, and *P. uechtritzii*, are described. The present study is the first report on the detailed chemical profiling and biological activities of the tested species. Thus, this work could provide valuable contributions to the scientific pool for the member of the genus *Prangos*. For each *Prangos* species, the chemical profile and biological activity depended on the extraction solvents used. Among these species, *P. meliocarpoides* var. *meliocarpoides* yielded the highest phenolic and flavonoid contents in the obtained extracts. Additionally, in terms of biological assays, *P. meliocarpoides* var. *meliocarpoides* displayed the most potent antioxidant activity as well as a significant inhibitory property on BChE, α-amylase, and tyrosinase, and these effects appear to be correlated to the presence of hydrolysable tannins. This research work presents valuable preliminary data on the three members of *Prangos* genus, displaying *P. meliocarpoides* var. *meliocarpoides* as the most promising one. However, further investigations such as in vivo bioavailability and toxicity studies need to be performed in the future, before projecting the plant for possible nutraceutical/functional food and/or pharmaceutical applications. 

## Figures and Tables

**Table 1:** Total bioactive compounds and total antioxidant capacity (by phosphomolybdenum assay) of the tested extracts (*n* = 3).

Species	Solvents	TPC (mg GAE/g)	TFC (mg RE/g)	PBD (mmol TE/g)
*P. heyniae*	Hexane	17.55 ± 0.16 ^g^	3.43 ± 0.14 ^j^	1.93 ± 0.02 ^b^
	EA	21.85 ± 0.36 ^f^	12.93 ± 0.35 ^f^	2.30 ± 0.10 ^a^
	MeOH	32.13 ± 0.76 ^c^	28.75 ± 0.36 ^b^	1.51 ± 0.11 ^de^
	Water	38.77 ± 0.01 ^b^	16.19 ± 0.24 ^d^	1.39 ± 0.03 ^ef^
*P. meliocarpoides* var. *meliocarpoides*	Hexane	22.63 ± 0.16 ^f^	8.19 ± 0.23 ^h^	2.52 ± 0.06 ^a^
	EA	26.15 ± 1.40 ^e^	19.40 ± 0.60 ^c^	2.49 ± 0.05 ^a^
	MeOH	40.03 ± 0.68 ^b^	44.66 ± 0.68 ^a^	1.82 ± 0.11 ^bc^
	Water	44.28 ± 0.27 ^a^	11.00 ± 0.18 ^g^	1.46 ± 0.02 ^def^
*P. uechtritzii*	Hexane	18.70 ± 0.08 ^g^	1.72 ± 0.07 ^k^	1.79 ± 0.04 ^bc^
	EA	25.45 ± 0.12 ^e^	5.72 ± 0.05 ^i^	2.35 ± 0.17 ^a^
	MeOH	31.20 ± 0.16 ^c^	28.22 ± 0.54 ^b^	1.67 ± 0.13 ^cd^
	Water	29.62 ± 0.07 ^d^	14.75 ± 0.30 ^e^	1.21 ± 0.03 ^f^

Values are reported as mean ± SD of three parallel experiments. EA: ethyl acetate; MeOH: methanol; TPC: total phenolic content; TFC: total flavonoid content; PBD: phosphomolybdenum; GAE: gallic acid equivalent; RE: rutin equivalent; TE: Trolox equivalent. Different letters indicate significant differences between the tested extracts (*p* < 0.05).

**Table 2:** Chemical composition of the tested extracts in positive and negative ion mode (mg/g).

M-H	Molecular Formula	Fragments	Name and References	*P. heyniae*- Hexane	*P. heyniae*- EA	*P. heyniae*- MeOH	*P. heyniae*- Water	*P. meliocarpoides*- Hexane	*P. meliocarpoides*-EA	*P. meliocarpoides*-MeOH	*P. meliocarpoides*-Water	*P. uechtritzii*-Hexane	*P. uechtritzii*-EA	*P. uechtritzii*-MeOH	*P. uechtritzii*-Water
			**Condensed Tannins**												
609.088050	C_29_H_21_O_15_	457 305 249	Epigallocatechin 3,5-di-gallate [38,39]	0.15 ± 0.01	0.08 ± 0.01	0.47 ± 0.02	0.89 ± 0.05	nd	nd	nd	nd	nd	nd	nd	nd
609.088050	C_29_H_21_O_15_	457 305 249	Epigallocatechin di-gallate isomer [38,39]	0.05 ± 0.01	0.03 ± 0.01	7.93 ± 0.05	3.21 ± 0.05	nd	nd	nd	nd	nd	nd	nd	nd
M + H															
763,151	C_37_H_31_O_1__8_	611 595 458 443 425 317 305 287	Galloylprodelphinidin isomer 1 [40]	nd	nd	2.33 ± 0.05	1.85 ± 0.04	nd	nd	nd	nd	nd	nd	nd	nd
761,1398	C_37_H_28_O_18_	611 595 458 443 425 317 305 287	Galloylprodelphinidin isomer 2 [40]	0.01 ± 0.01	0.01 ± 0.01	0.31 ± 0.03	0.25 ± 0.02	nd	nd	nd	nd	nd	nd	nd	nd
763,1505	C_37_H_30_O_18_	611 595 458 443 425 317 305 287	Galloylprodelphinidin isomer 3 [40]	0.01 ± 0.01	nd	4.66 ± 0.05	5.79 ± 0.07	nd	nd	nd	nd	nd	nd	nd	nd
761,1398	C_37_H_28_O_18_	611 595 458 443 425 317 305 287	Galloylprodelphinidin isomer 4 [40]	nd	nd	3.22 ± 0.05	5.47 ± 0.09	nd	nd	nd	nd	nd	nd	nd	nd
915,162	C_44_H_34_O_22_	611 595 458 443 425 317 305 287	di-*O*-galloylprodelphinidin dimer isomer 1 [40]	0.01 ± 0.01	nd	3.11 ± 0.05	2.81 ± 0.06	0.34 ± 0.02	0.23 ± 0.02	0.48 ± 0.01	0.11 ± 0.01	nd	nd	0.22 ± 0.03	0.24 ± 0.02
915,1615	C_44_H_34_O_22_	611 595 458 443 425 317 305 287	di-*O*-galloylprodelphinidin dimer isomer 2 [40]	nd	nd	nd	nd	nd	nd	nd	nd	nd	nd	nd	nd
459,0925	C_23_H_20_O_10_	303,0579	Epigallocatechin-gallate * [41]	nd	nd	nd	nd	nd	nd	nd	nd	nd	5.09 ± 0.08	53.52 ± 0.22	31.25 ± 0.44
345,0614	C_17_H_13_O_8_	315	Methylellagic acid * [42,43]	nd	nd	1.62 ± 0.05	1.27 ± 0.05	0.93 ± 0.04	1.86 ± 0.04	2.43 ± 0.04	2.04 ± 0.02	nd	nd	nd	nd
357.0461	C_14_H_12_O_1__1_		Chebulic acid [44]	nd	nd	nd	nd	0.02 ± 0.01	0.04 ± 0.02	0.05 ± 0.01	0.88 ± 0.01	nd	nd	nd	nd
357.046	C_14_H_12_O_11_		Chebulic acid isomer 1 [44]	nd	nd	nd	nd	nd	0.02 ± 0.01	0.01 ± 0.01	1.71 ± 0.03	nd	nd	nd	nd
761,136	C_37_H_30_O_18_	593 423	(epi)gallocatechin-gallocatechin-gallate dimer [39,45]	nd	0.56 ± 0.02	4.81 ± 0.05	2.63 ± 0.05	nd	nd	nd	nd	nd	nd	nd	nd
761,136	C_37_H_30_O_18_	593 423	(epi)gallocatechin-gallocatechin-gallate dimer [39,45]	nd	0.35 ± 0.01	3.00 ± 0.05	1.63 ± 0.05	nd	nd	nd	nd	nd	nd	nd	nd
761,136	C_37_H_30_O_18_	593 423	(epi)gallocatechin-gallocatechin-gallate dimer [39,45]	nd	3.12 ± 0.06	23.54 ± 0.25	12.4 ± 0.11	nd	nd	nd	nd	nd	nd	nd	nd
761,136	C_37_H_30_O_18_	593 423	(epi)gallocatechin-gallocatechin-gallate dimer [39,45]	nd	2.22 ± 0.05	13.64 ± 0.11	7.52 ± 0.11	nd	nd	nd	nd	nd	nd	nd	nd
745,141	C_37_H_30_O_17_		Epicatechin-(4beta- > 8)-epigallocatechin 3′-gallate [39,45]	nd	0.59 ± 0.06	7.31 ± 0.07	4.48 ± 0.10	nd	nd	nd	nd	nd	nd	nd	nd
913,1468	C_44_H_33_O_22_	761 423	(epi)gallocatechin-gallate dimer [39,45]	nd	3.46 ± 0.11	18.02 ± 0.11	12.39 ± 0.22	nd	nd	nd	nd	nd	nd	nd	nd
913,1468	C_44_H_33_O_22_	761 423	(epi)gallocatechin-gallate dimer [39,45]	nd	1.85 ± 0.09	9.71 ± 0.11	8.46 ± 0.10	nd	nd	nd	nd	nd	nd	nd	nd
745,141	C_37_H_30_O_18_		Epicatechin-(4beta- > 8)-epigallocatechin 3′-gallate [39,45]	nd	0.40 ± 0.05	5.79 ± 0.08	6.57 ± 0.05	nd	nd	nd	nd	nd	nd	nd	nd
745,141	C_37_H_30_O_19_		Epicatechin-(4beta- > 8)-epigallocatechin 3′-gallate [39,45]	nd	0.86 ± 0.05	5.89 ± 0.09	1.96 ± 0.02	nd	nd	nd	nd	nd	nd	nd	nd
915,162	C_44_H_36_O_2__2_	457	Assamicain [46]	nd	0.17 ± 0.05	3.52 ± 0.22	4.58 ± 0.04	nd	nd	nd	nd	nd	nd	nd	nd
457,0771	C_22_H_17_O_11_	331 305 169	gallocatechin gallate* [46]	0.01 ± 0.01	1.61 ± 0.05	7.23 ± 0.09	7.32 ± 0.05	nd	nd	nd	nd	nd	nd	nd	nd
457,0771	C_22_H_17_O_11_	331 305 169	epi-gallocatechin gallate*	nd	1.77 ± 0.05	8.61 ± 0.11	6.61 ± 0.09	nd	nd	nd	nd	nd	nd	nd	nd
915,162	C_44_H_36_O_22_	457	Assamicain B [46]	nd	0.05 ± 0.01	7.24 ± 0.09	5.73 ± 0.07	nd	nd	nd	nd	nd	nd	nd	nd
			***TOTAL***	0.24	17.12	141.95	103.77	1.29	2.15	2.97	4.73	nd	5.09	53.74	31.49
	M-H		**Hydrolisable tannins**												
801,1135	C_35_H_29_O_23_		tetragalloylquinic acid [39,47,48,49]	nd	nd	0.99 ± 0.05	1.51 ± 0.09	0.26 ± 0.03	0.21 ± 0.01	1.33 ± 0.06	3.02 ± 0.06	nd	nd	nd	nd
618,0935	C_27_H_23_O_17_		3-*O*-Galloyl-4,6-(S)-HHDP-d-glucose [39,47,48,49]	nd	nd	nd	nd	0.19 ± 0.02	0.16 ± 0.03	13.35 ± 0.36	6.11 ± 0.06	nd	nd	nd	nd
787,0994	C_34_H_27_O_22_	617 321	1,3-Digalloyl-4,6-HHDP-glucose/Heterophyliin A [39,47,48,49]	nd	nd	nd	nd	1.28 ± 0.04	0.01 ± 0.01	26.06 ± 0.35	12.48 ± 0.12	nd	nd	nd	nd
787,1152	C_35_H_30_O_21_		2″,3″,6″-Tris-*O*-(3,4,5-trihydroxybenzoyl)-3′-Glucosyl-2′,4′,6′-trihydroxyacetophenone [39,47,48,49]	nd	nd	1.71 ± 0.06	1.16 ± 0.06	0.22 ± 0.03	0.15 ± 0.03	37.97 ± 0.99	18.84 ± 0.17	nd	nd	nd	nd
787,0994	C_34_H_27_O_22_	617 321	Heterophylliin A isomer	nd	nd	1.86 ± 0.05	1.16 ± 0.09	0.47 ± 0.05	nd	36.29 ± 0.85	22.62 ± 0.23	nd	nd	nd	nd
801,155	C_35_H_29_O_22_		tetragalloylquinic acid [39,47,48,49]	nd	nd	nd	nd	8.58 ± 0.06	0.36 ± 0.03	18.63 ± 0.88	12.13 ± 0.21	nd	nd	nd	nd
1237,71	C_55_H_34_O_34_		Prostratin A or Tirucallin A	nd	nd	nd	nd	0.11 ± 0.02	0.04 ± 0.02	0.32 ± 0.01	2.11 ± 0.06	nd	nd	nd	nd
1083.0581	C_48_H_27_O_30_		alfa-Punicalagin * [48]	nd	nd	nd	nd	4.26 ± 0.14	8.82 ± 0.11	0.11 ± 0.01	0.65 ± 0.05	nd	nd	nd	nd
1083.0581	C_48_H_27_O_30_		beta-punicalagin * [48]	nd	nd	nd	nd	9.12 ± 0.16	14.64 ± 0.23	0.13 ± 0.01	1.13 ± 0.02	nd	nd	nd	nd
1083.0581	C_48_H_27_O_300_	601	Punicalagin derivative [48]	nd	nd	nd	nd	4.72 ± 0.22	8.40 ± 0.22	0.32 ± 0.01	4.93 ± 0.09	nd	nd	nd	nd
1083.0581	C_48_H_28_O_30_	601	Terchebuline [39,45,48]	nd	nd	nd	nd	8.71 ± 0.03	17.53 ± 0.85	nd	nd	nd	nd	nd	nd
637.1052	C_27_H_24_O_18_	483 465 313	trigalloyl glucose [39,47,48,49]	nd	nd	nd	nd	0.11 ± 0.03	0.23 ± 0.09	1.32 ± 0.02	3.67 ± 0.04	nd	nd	nd	nd
637.1048	C_27_H_24_O_18_	483 465 313	trigalloyl glucose [39,47,48,49]	nd	nd	nd	nd	0.04 ± 0.01	0.07 ± 0.03	1.43 ± 0.04	4.48 ± 0.04	nd	nd	nd	nd
637.1048	C_27_H_24_O_18_	483 465 313	trigalloyl glucose [39,47,48,49]	nd	nd	nd	nd	0.08 ± 0.01	0.13 ± 0.02	0.37 ± 0.02	27.63 ± 0.34	nd	nd	nd	nd
971.1733	C_43_H_38_O_26_	817 635 465	tetragalloyl-syrigylglucose [39,47,48,49]	nd	nd	nd	nd	0.01 ± 0.01	0.07 ± 0.03	3.43 ± 0.08	4.37 ± 0.06	nd	nd	nd	nd
637.1045	C_27_H_24_O_18_		trigalloyl glucose [39,47,48,49]	nd	nd	nd	nd	0.46 ± 0.04	1.35 ± 0.05	7.63 ± 0.09	28.29 ± 0.33	nd	nd	nd	nd
939.1110	C_41_H_30_O_26_		Tellimagrandin II [47,48,49,50]	nd	nd	nd	nd	0.05 ± 0.02	0.04 ± 0.01	4.77 ± 0.08	0.19 ± 0.01	nd	nd	nd	nd
989.1483	C_42_H_36_O_28_		Methyl neochebulinate [44]	nd	nd	nd	nd	0.04 ± 0.03	0.11 ± 0.03	10.02 ± 0.33	2.07 ± 0.06	nd	nd	nd	nd
787,0997	C_34_H_27_O_22_		tetragalloyl glucose [39,47,48,49]	nd	nd	nd	nd	0.48 ± 0.05	0.66 ± 0.03	11.15 ± 0.34	32.53 ± 0.22	nd	nd	nd	nd
955	C_41_H_32_O_27_		Chebulinic acid [44,47,48]	nd	nd	nd	nd	2.02 ± 0.07	2.55 ± 0.09	13.5 ± 0.08	53.83 ± 0.76	nd	nd	nd	nd
1001.2199	C_45_H_45_O_26_	909 617 465	digalloyl-dishikimoyl-synapoylglucose [39,45,47,49]	nd	nd	nd	nd	nd	nd	0.55 ± 0.01	nd	nd	nd	nd	nd
1001.2199	C_45_H_45_O_26_	910 617 465	digalloyl-dishikimoyl-synapoylglucose [39,45,47,49]	nd	nd	nd	nd	nd	nd	1.54 ± 0.05	nd	nd	nd	nd	nd
1001.2199	C_45_H_45_O_26_	911 617 465	digalloyl-dishikimoyl-synapoylglucose [39,45,47,49]	nd	nd	nd	nd	nd	nd	0.74 ± 0.01	nd	nd	nd	nd	nd
1001.2199	C_45_H_45_O_26_	912 617 465	digalloyl-dishikimoyl-synapoylglucose [39,45,47,49]	nd	nd	nd	nd	nd	nd	0.37 ± 0.01	nd	nd	nd	nd	nd
987.1887	C_43_H_40_O_26_	799 771 617 465 313	trigalloyl-syrigyl-syìhykimil-glucose [39,45,47,49]	nd	nd	nd	nd	0.11 ± 0.02	nd	12.29 ± 0.08	10.84 ± 0.07	nd	nd	nd	nd
1001.2199	C_45_H_45_O_26_	913 617 465	digalloyl-dishikimoyl-synapoylglucose [39,45,47,49]	nd	nd	nd	nd	nd	nd	0.42 ± 0.03	nd	nd	nd	nd	nd
			***TOTAL***	nd	nd	4.56	3.82	41.29	55.52	204.04 ± 1.52	251.9 ± 0.99	nd	nd	nd	nd
M + H			**Coumarins**												
177,0552	C_10_H_9_O_3_	133	8-methoxy coumarin * [8,51]	0.05 ± 0.01	0.03 ± 0.01	0.86 ± 0.05	0.84 ± 0.04	0.19 ± 0.02	0.23 ± 0.03	5.83 ± 0.11	1.04 ± 0.02	0.05 ± 0.01	0.09 ± 0.01	14.39 ± 0.11	16.12 ± 0.33
327,0896	C_18_H_15_O_6_		4-coumaroyl-4′-hydroxyphenyllactate	0.02 ± 0.01	0.01 ± 0.01	0.51 ± 0.03	0.87 ± 0.05	nd	nd	nd	nd	0.02 ± 0.01	0.01 ± 0.01	3.88 ± 0.07	2.38 ± 0.06
287,1016	C_16_H_15_O_5_	269 245 201	heraclenin/prangenin * [8,51]	nd	nd	2.07 ± 0.11	4.14 ± 0.09	nd	nd	nd	nd	nd	nd	nd	nd
247,097	C_14_H_14_O_4_	188 146	8-(2′,3′-dihydroxyisopentyl)-7-hydroxycoumarin /marmesin* [8,51]	nd	nd	0.74 ± 0.05	3.25 ± 0.09	0.52 ± 0.03	nd	2.41 ± 0.04	7.06 ± 0.04	nd	nd	4.71 ± 0.06	5.87 ± 0.11
271,091	C_16_H_13_O_4_	203 175	Imperatorin* [8,51]	nd	nd	nd	nd	0.25 ± 0.03	0.21 ± 0.03	8.28 ± 0.11	14.72 ± 0.33	nd	nd	nd	nd
327,166	C_20_H_23_O_4_		Dentatin * [8,51]	nd	nd	nd	nd	nd	nd	nd	nd	4.05 ± 0.03	1.54 ± 0.03	2.61 ± 0.05	3.68 ± 0.11
			***TOTAL***	0.07	0.04	4.18	9.11	0.94	0.43	16.51	22.82	4.12	1.64	25.59	28.05
M-H			**Flavonoid derivatives**												
785,162	C_36_H_32_O_20_		Degalloyltheaflavonin	nd	nd	nd	nd	2.04 ± 0.03	2.65 ± 0.11	2.17 ± 0.09	3.16 ± 0.22	nd	nd	nd	nd
771,2372	C_34_H_42_O_20_	625,1824 479,0928 317,0729	Typhaneoside	nd	nd	nd	nd	1.37 ± 0.05	1.05 ± 0.08	30.82 ± 0.55	15.9 ± 0.78	nd	nd	nd	nd
433,1171	C_21_H_21_O_10_		Apigenin-C-hexoside	nd	nd	nd	nd	1.26 ± 0.09	0.01 ± 0.01	0.02 ± 0.01	0.28 ± 0.02	nd	nd	nd	nd
601,1193	C_28_H_25_O_15_	431 329	2′-*O*-Galloylquercitrin	nd	nd	nd	nd	1.25 ± 0.02	1	29.98 ± 0.66	14.72 ± 0.22	nd	nd	nd	nd
585,1269	C_28_H_25_O_14_	431 415 285	Kaempferol-deoxyhexoside-gallate [39,45,49]	nd	nd	nd	nd	0.12 ± 0.01	0.32 ± 0.03	19.61 ± 0.44	9.87 ± 0.08	nd	nd	nd	nd
769.2205	C_34_H_42_O_20_	605 314	Isorhamnetin-7-*O*-hexoside-3-*O*-hexosidedeoxyhexoside [39,45,49]	nd	nd	nd	nd	nd	nd	nd	nd	nd	nd	0.24 ± 0.02	0.11 ± 0.01
625.1198	C_30_H_26_O_15_	448 301	Quercetin-7-*O*-hexoside-caffeoyl [39,45,49]	nd	nd	nd	nd	nd	nd	nd	nd	nd	nd	3.08 ± 0.09	1.4 ± 0.02
625.1196	C_30_H_26_O_15_	449 301	Quercetin-7-*O*-hexoside-caffeoyl isomer [39,45,49]	nd	nd	nd	nd	nd	nd	nd	nd	nd	nd	0.94 ± 0.06	0.25 ± 0.01
479.0815	C_21_H_20_O_13_	316 271 179	Myricetin-3-*O*-glucopyranoside *	nd	nd	nd	nd	nd	nd	nd	nd	nd	nd	4.52 ± 0.09	0.71 ± 0.02
769.2204	C_34_H_42_O_20_	605 314	Isorhamnetin-7-*O*-hexoside-3-*O*-hexosidedeoxyhexoside [39,45,49]	nd	nd	nd	nd	nd	nd	nd	nd	nd	nd	1.54 ± 0.11	0.71 ± 0.03
769.2200	C_34_H_42_O_20_	605 314	Isorhamnetin-7-*O*-hexoside-3-*O*-hexosidedeoxyhexoside [39,45,49]	nd	nd	nd	nd	nd	nd	nd	nd	nd	nd	0.78 ± 0.08	0.43 ± 0.01
609.1464	C_27_H_30_O_16_	301 271 179	Rutin *	nd	nd	nd	nd	nd	nd	nd	nd	nd	nd	8.55 ± 0.11	2.75 ± 0.11
463.0884	C_21_H_20_O_12_	316 271	Myricetin-3-*O*-rhamnopyranoside *	nd	nd	nd	nd	nd	nd	nd	nd	nd	nd	11.7 ± 0.22	3.07 ± 0.11
463.0887	C_21_H_20_O_12_	316 271	Myricetin-7-*O*-rhamnopyranoside *	nd	nd	nd	nd	nd	nd	nd	nd	nd	nd	7.37 ± 0.13	1.11 ± 0.22
623.1627	C_28_H_32_O_16_	461 314 315 299	Isorhamnetin-7-*O*-rhamnopyranosyl-3-*O*-glucopyranoside *	nd	nd	nd	nd	nd	nd	nd	nd	nd	nd	5.51 ± 0.22	1.86 ± 0.22
463.0969	C_21_H_20_O_12_	301	Quercetin-3-*O*-galactopyranoside *	nd	nd	nd	nd	0.06 ± 0.03	0.16 ± 0.03	1.05 ± 0.02	3.69 ± 0.05	nd	nd	nd	nd
477.0684	C_21_H_18_O_13_	301 271	Quercetin-3-*O*-glucuronide *	nd	nd	nd	nd	nd	nd	nd	nd	nd	nd	16.33 ± 0.99	10.17 ± 0.22
623.1638	C_28_H_32_O_16_	315 300	Isorhamnetin-3-*O*-hexosyl-deoxyhexoside [39,45,49]	nd	nd	nd	nd	nd	nd	nd	nd	nd	nd	2.18 ± 0.11	0.73 ± 0.05
623.1622	C_28_H_32_O_16_	315 300	Isorhamnetin-3-*O*-rutinoside *	nd	nd	nd	nd	nd	nd	nd	nd	nd	nd	5.05 ± 0.09	1.61 ± 0.07
477.1054	C_22_H_22_O_12_	314 300 271 255	Isorhamnetin-3-*O*-glucopyranoside *	nd	nd	nd	nd	nd	nd	nd	nd	nd	nd	2.02 ± 0.05	1.4 ± 0.07
463.0854	C_21_H_20_O_12_	301 271 255 179 151	Quercetin-3-*O*-glucopyranoside *	nd	nd	nd	nd	nd	nd	nd	nd	nd	nd	34.3 ± 0.51	10.57 ± 0.33
477.1043	C_22_H_22_O_12_	314 300 271 255	Isorhamnetin-3-*O*-galactopyranoside *	nd	nd	nd	nd	nd	nd	nd	nd	nd	nd	10.03 ± 0.11	3.11 ± 0.05
491.0840	C_22_H_20_O_13_	315 300 271 255	Isorhamnetin-3-*O*-glucuronide *	nd	nd	nd	nd	nd	nd	nd	nd	nd	nd	11.93 ± 0.22	6.18 ± 0.07
431.0978	C_21_H_20_O_10_	285	Kaempferol-3-*O*-rhamnoside *	nd	nd	nd	nd	nd	nd	nd	nd	nd	nd	8.18 ± 0.08	1.71 ± 0.06
461.1083	C_22_H_21_O_11_	314 300	Isorhamnetin-7-*O*-rhamnopyranoside *	nd	nd	nd	nd	nd	nd	nd	nd	nd	nd	4.12 ± 0.11	1.06 ± 0.05
			***TOTAL***	nd	nd	nd	nd	6.10 ± 0.05	5.19 ± 0.09	83.65 ± 0.99	47.62 ± 0.08	nd	2.68 ± 0.03	138.03 ± 1.25	48.92 ± 0.66
M + H			**Hydroxycinnamic derivatives**									nd			
339,11	C_16_H_19_O_8_	191	1-coumaroyl quinic acid [39,45,49]	1.83 ± 0.01	5.48 ± 0.07	8.52 ± 0.05	6.32 ± 0.07	0.13 ± 0.01	1.44 ± 0.07	3.06 ± 0.04	0.75 ± 0.05	nd	nd	4.11 ± 0.04	3.91 ± 0.04
M-H												nd	nd		
341,0873	C_15_H_17_O_9_	179	Caffeoyl hexose [39,45,49]	nd	0.16 ± 0.05	0.76 ± 0.06	1.31 ± 0.09	0.01 ± 0.01	0.02 ± 0.01	0.01 ± 0.01	0.02 ± 0.01	nd	nd	0.08 ± 0.01	0.23 ± 0.02
501,1619	C_22_H_29_O_13_	417 399 285 152	Clemomandshuricoside B	nd	nd	nd	nd	nd	nd	nd	nd	nd	nd	2.09 ± 0.06	0.55 ± 0.02
			***TOTAL***	1.83	5.64	9.26 ± 0.10	7.61 ± 0.09	0.14 ± 0.05	1.46 ± 0.06	3.07 ± 0.05	0.77 ± 0.03	nd	nd	6.27 ± 0.07	4.69 ± 0.02
M + H			**Other compounds**									nd	nd		
225,1399	C_13_H_20_O_3_	147 119 103 79 88	6-hydroxy-3-oxo-alpha-ionol	3.72 ± 0.01	3.12 ± 0.05	10.2 ± 0.20	10.8 ± 0.11	nd	0.89 ± 0.03	5.05 ± 0.11	0.08 ± 0.01	nd	nd	6.52 ± 0.05	6.57 ± 0.02
381,1658	C_16_H_29_O_10_	364 219 200	Prenyl arabinosyl-(1- > 6)-glucoside	0.11 ± 0.01	0.27 ± 0.05	7.36 ± 0.11	7.92 ± 0.10	nd	nd	nd	nd	nd	nd	3.25 ± 0.05	3.61 ± 0.04
787,1152	C_35_H_31_O_31_		2″,3″,6″-Tris-*O*-(3,4,5-trihydroxybenzoyl)-3′-Glucosyl-2′,4′,6′-trihydroxyacetophenone	nd	nd	1.71 ± 0.06	1.16 ± 0.05	0.21 ± 0.05	0.15 ± 0.03	37.97 ± 0.54	18.84 ± 0.22	nd	nd	nd	nd
285,0974	C_13_H_17_O_7_		p-hydroxy-benzoic acid rhamnosyl ester	4.92 ± 0.02	3.83 ± 0.06	3.52 ± 0.09	3.65 ± 0.09	nd	nd	nd	nd	0.57 ± 0.03	2.54 ± 0.05	5.46 ± 0.11	2.91 ± 0.03
315.1088	C_14_H_18_O_8_	167	glucovanillin	nd	nd	2.98 ± 0.09	2.27 ± 0.11	nd	nd	nd	nd	2.21 ± 0.09	6.78 ± 0.09	10.27 ± 0.22	3.67 ± 0.05
			***TOTAL***	8.73	7.22	25.77	25.8	0.2	1.04	43.02	18.92	2.78	9.32	25.5	16.75

nd: not detected; * identity confirmed by injection of reference compound.

**Table 3:** Antioxidant properties of the tested extracts (*n* = 3).

Species	Solvents	DPPH (mg TE/g)	ABTS (mg TE/g)	CUPRAC (mg TE/g)	FRAP (mg TE/g)	MCA (mg EDTAE/g)
*P. heyniae*	Hexane	5.42 ± 0.37 ^g^	na	35.97 ± 1.80 ^h^	20.27 ± 0.55 ^i^	27.34 ± 0.34 ^a^
	EA	10.15 ± 0.47 ^e^	10.44 ± 0.91 ^g^	59.74 ± 0.63 ^ef^	31.60 ± 0.62 ^gh^	26.18 ± 0.16 ^ab^
	MeOH	46.65 ± 0.34 ^c^	60.98 ± 0.04 ^d^	108.43 ± 1.52 ^c^	70.21 ± 3.22 ^d^	15.53 ± 0.90 ^e^
	Water	50.66 ± 0.42 ^b^	90.74 ± 1.35 ^a^	129.89 ± 3.24 ^b^	93.14 ± 1.75 ^b^	27.79 ± 0.32 ^a^
*P. meliocarpoides* var. *meliocarpoides*	Hexane	4.13 ± 0.33 ^g^	8.90 ± 0.95 ^g^	55.98 ± 0.84 ^f^	28.61 ± 1.00 ^h^	26.38 ± 0.15 ^ab^
	EA	9.70 ± 0.09 ^e^	17.72 ± 0.08 ^f^	62.26 ± 1.02 ^e^	34.22 ± 0.55 ^g^	19.91 ± 0.22 ^cd^
	MeOH	52.27 ± 0.28 ^a^	77.88 ± 1.07 ^b^	133.19 ± 1.09 ^b^	80.79 ± 1.03 ^c^	18.55 ± 0.61 ^d^
	Water	52.01 ± 0.52 ^ab^	92.84 ± 0.44 ^a^	154.04 ± 2.10 ^a^	104.34 ± 1.07 ^a^	21.17 ± 0.54 ^c^
*P. uechtritzii*	Hexane	2.18 ± 0.62 ^h^	17.00 ± 0.74 ^f^	35.91 ± 1.32 ^h^	21.79 ± 0.85 ^i^	18.50 ± 1.92 ^d^
	EA	8.11 ± 0.35 ^f^	26.29 ± 0.44 ^e^	45.62 ± 2.18 ^g^	27.99 ± 1.69 ^h^	20.75 ± 0.25 ^c^
	MeOH	34.55 ± 0.63 ^d^	75.03 ± 0.43 ^c^	96.69 ± 2.56 ^d^	56.31 ± 0.36 ^f^	17.88 ± 0.83 ^d^
	Water	34.52 ± 0.96 ^d^	74.74 ± 0.58 ^c^	95.43 ± 0.80 ^d^	65.86 ± 0.82 ^e^	24.57 ± 0.40 ^b^

Values are reported as mean ± SD of three parallel experiments. EA: ethyl acetate; MeOH: methanol; TE: Trolox equivalent; EDTAE: EDTA equivalents. na: not active. Different letters in the same column indicate significant differences between the tested extracts (*p* < 0.05).

**Table 4:** Enzyme inhibitory effects of the tested extracts (*n* = 3).

Species	Solvents	AChE (mg GALAE/g)	BChE (mg GALAE/g)	Tyrosinase (mg KAE/g)	Amylase (mmol ACAE/g)	Glucosidase (mmol ACAE/g)
*P. heyniae*	Hexane	2.39 ± 0.06 ^a^	7.83 ± 0.18 ^a^	56.07 ± 1.46 ^ef^	0.36 ± 0.01 ^c^	0.67 ± 0.04 ^bc^
	EA	1.58 ± 0.38 ^cd^	7.64 ± 0.15 ^ab^	54.21 ± 1.32 ^f^	0.41 ± 0.01 ^b^	0.62 ± 0.04 ^cd^
	MeOH	2.36 ± 0.18 ^a^	4.28 ± 0.16 ^c^	65.20 ± 0.89 ^c^	0.17 ± 0.01 ^e^	0.46 ± 0.08 ^e^
	Water	0.35 ± 0.08 ^e^	na	17.34 ± 0.38 ^i^	0.06 ± 0.01 ^g^	na
*P. meliocarpoides* var. *meliocarpoides*	Hexane	1.16 ± 0.27 ^d^	7.97 ± 0.06 ^a^	81.15 ± 0.19 ^a^	0.46 ± 0.01 ^a^	0.61 ± 0.02 ^cd^
	EA	na	7.32 ± 0.80 ^ab^	59.92 ± 0.96 ^d^	0.40 ± 0.02 ^b^	0.56 ± 0.01 ^d^
	MeOH	na	3.34 ± 0.46 ^d^	70.57 ± 0.59 ^b^	0.21 ± 0.01 ^d^	0.74 ± 0.01 ^ab^
	Water	0.19 ± 0.01 ^e^	na	21.23 ± 1.33 ^h^	0.05 ± 0.01 ^g^	na
*P. uechtritzii*	Hexane	2.34 ± 0.12 ^ab^	7.63 ± 0.39 ^ab^	58.77 ± 1.76 ^de^	0.39 ± 0.01 ^b^	0.64 ± 0.01 ^cd^
	EA	2.13 ± 0.18 ^ab^	6.91 ± 0.17 ^b^	61.03 ± 1.10 ^d^	0.40 ± 0.01 ^b^	0.59 ± 0.01 ^cd^
	MeOH	1.76 ± 0.14 ^bc^	1.58 ± 0.12 ^e^	68.03 ± 0.39 ^bc^	0.20 ± 0.01 ^d^	0.78 ± 0.01 ^a^
	Water	na	0.34 ± 0.04 ^f^	27.54 ± 1.03 ^g^	0.09 ± 0.01 ^f^	na

Values are reported as mean ± SD of three parallel experiments. EA: Ethyl acetate; MeOH: Methanol; GALAE: Galantamine equivalent; KAE: Kojic acid equivalent; ACAE: Acarbose equivalent; na: not active. Different letters in the same column indicate significant differences between the tested extracts (*p* < 0.05).

## Data Availability

The data are contained within the article and supplementary materials.

## Supplementary Materials

The following supporting information can be downloaded at https://www.mdpi.com/article/10.3390/antiox11091712/s1, Figure S1: Pie charts summarising the relative percentage of phytochemicals identified and quantified in P. heyniae extracts; Figure S2: Pie charts summarising the relative percentage of phytochemicals identified and quantified in P. meliocarpoides extracts; Figure S3: Pie charts summarising the relative percentage of phytochemicals identified and quantified in P.uechtritzii extracts; Figure S4: MSn spectra showing the loss of water and proposed fragmentation for the peak assigned to chebulic acid; Figure S5: MSn spectra showing the loss of water and proposed fragmentation for the peak assigned to chebulic acid.
