# Supplementary material for: Phytochemical Screening, Antioxidant, and Enzyme Inhibitory Properties of Three Prangos Species (P. heyniae, P. meliocarpoides var. meliocarpoides, and P. uechtritzii) Depicted by Comprehensive LC-MS and Multivariate Data Analysis
Source: Antioxidants (Basel). 2022 Aug 30;11(9):1712. doi: 10.3390/antiox11091712 (PMC9495836; doi:10.3390/antiox11091712)
Supplement: Supplementary file 1 [file antioxidants-11-01712-s001.zip › antioxidants-1843286-supplementary.pptx]

## Slide 1
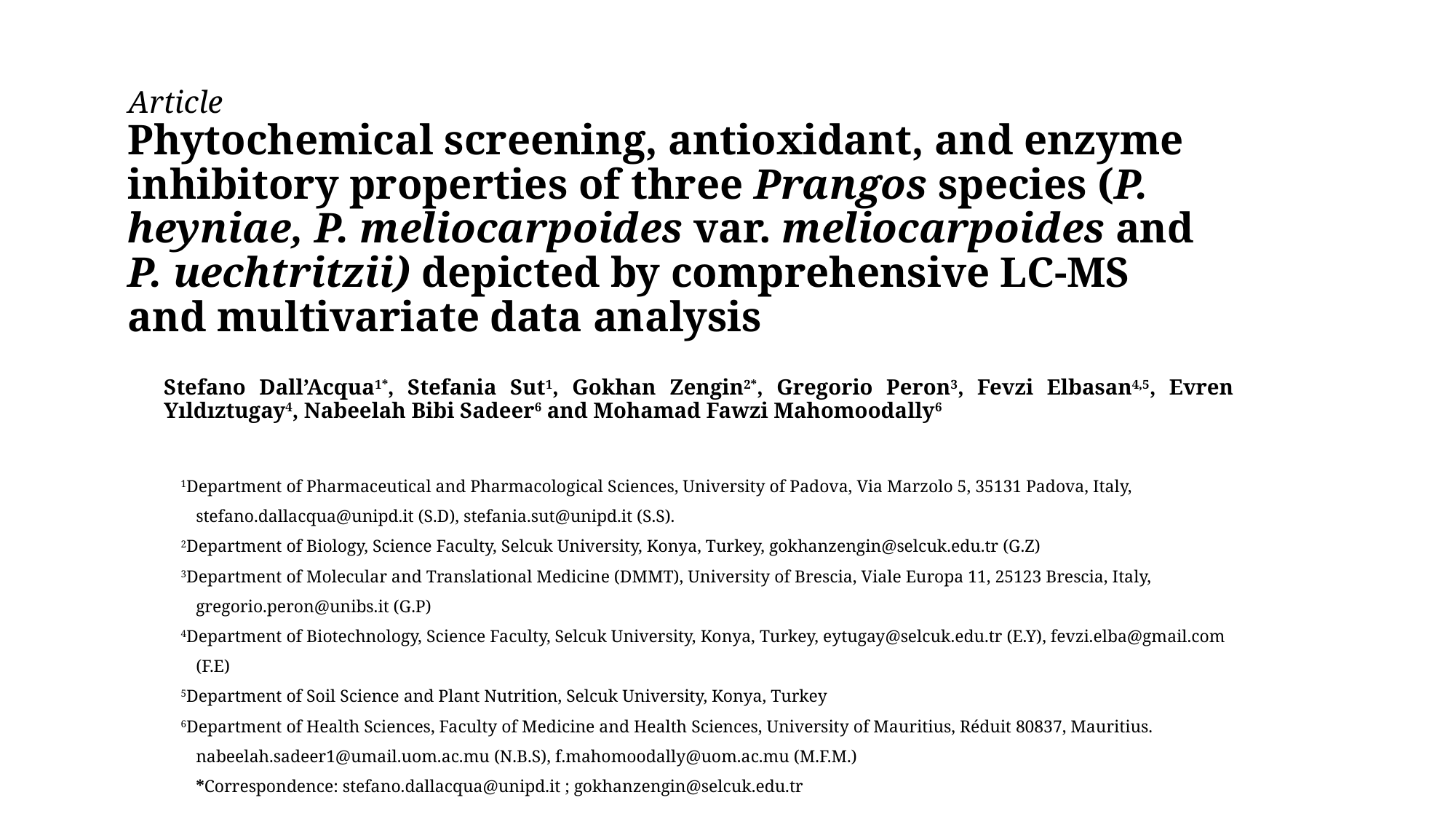

# Article Phytochemical screening, antioxidant, and enzyme inhibitory properties of three Prangos species (P. heyniae, P. meliocarpoides var. meliocarpoides and P. uechtritzii) depicted by comprehensive LC-MS and multivariate data analysis
Stefano Dall’Acqua1*, Stefania Sut1, Gokhan Zengin2*, Gregorio Peron3, Fevzi Elbasan4,5, Evren Yıldıztugay4, Nabeelah Bibi Sadeer6 and Mohamad Fawzi Mahomoodally6
1Department of Pharmaceutical and Pharmacological Sciences, University of Padova, Via Marzolo 5, 35131 Padova, Italy, stefano.dallacqua@unipd.it (S.D), stefania.sut@unipd.it (S.S).
2Department of Biology, Science Faculty, Selcuk University, Konya, Turkey, gokhanzengin@selcuk.edu.tr (G.Z)
3Department of Molecular and Translational Medicine (DMMT), University of Brescia, Viale Europa 11, 25123 Brescia, Italy, gregorio.peron@unibs.it (G.P)
4Department of Biotechnology, Science Faculty, Selcuk University, Konya, Turkey, eytugay@selcuk.edu.tr (E.Y), fevzi.elba@gmail.com (F.E)
5Department of Soil Science and Plant Nutrition, Selcuk University, Konya, Turkey
6Department of Health Sciences, Faculty of Medicine and Health Sciences, University of Mauritius, Réduit 80837, Mauritius. nabeelah.sadeer1@umail.uom.ac.mu (N.B.S), f.mahomoodally@uom.ac.mu (M.F.M.)
*Correspondence: stefano.dallacqua@unipd.it ; gokhanzengin@selcuk.edu.tr

## Slide 2
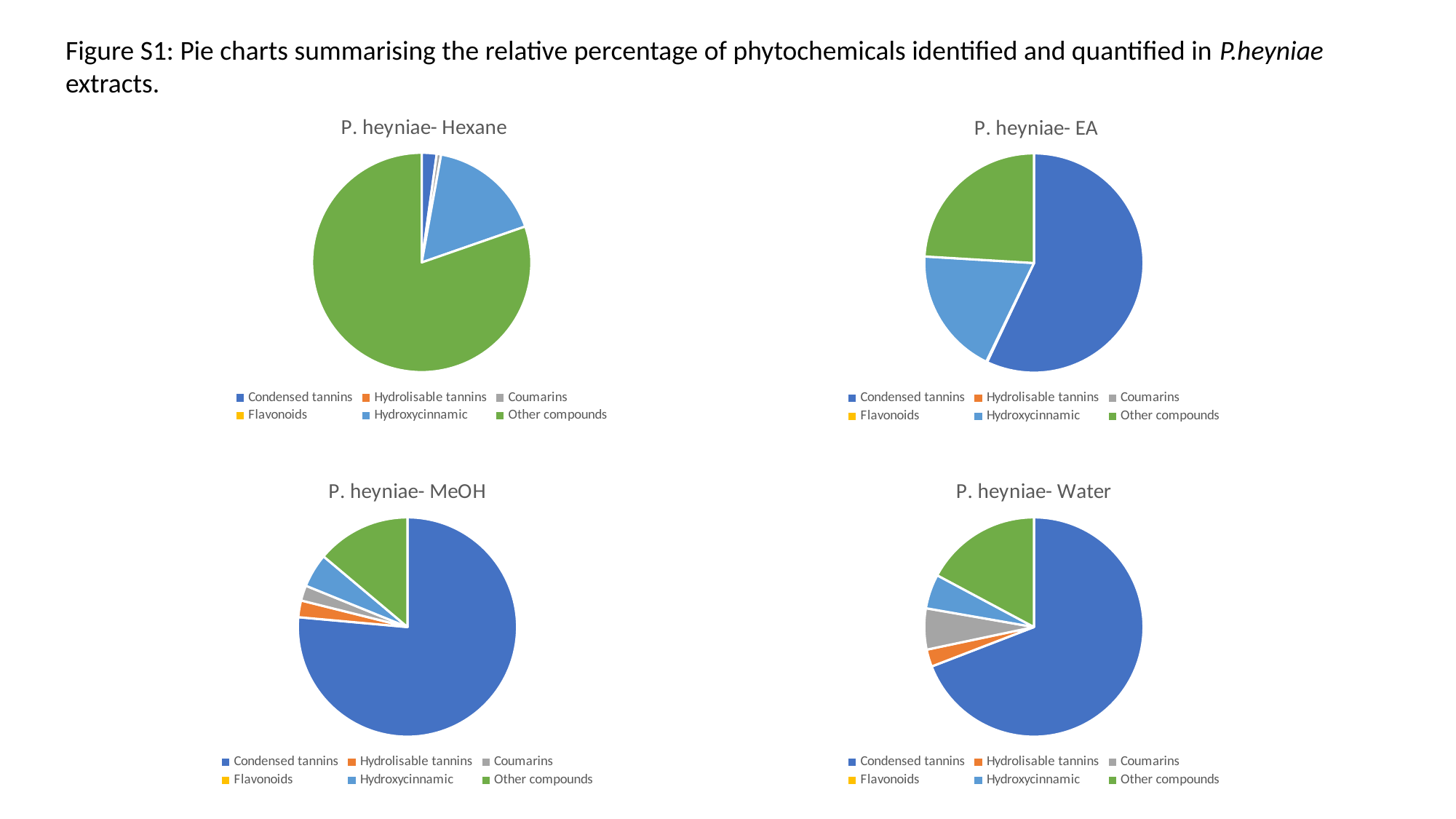

Figure S1: Pie charts summarising the relative percentage of phytochemicals identified and quantified in P.heyniae extracts.
### Chart:
| Category | P. heyniae- Hexane |
|---|---|
| Condensed tannins | 0.24000000000000005 |
| Hydrolisable tannins | 0.0 |
| Coumarins | 0.07 |
| Flavonoids | 0.0 |
| Hydroxycinnamic | 1.83 |
| Other compounds | 8.73 |
### Chart:
| Category | P. heyniae- EA |
|---|---|
| Condensed tannins | 17.12 |
| Hydrolisable tannins | 0.0 |
| Coumarins | 0.04 |
| Flavonoids | 0.0 |
| Hydroxycinnamic | 5.640000000000001 |
| Other compounds | 7.220000000000001 |
### Chart:
| Category | P. heyniae- MeOH |
|---|---|
| Condensed tannins | 141.95 |
| Hydrolisable tannins | 4.5600000000000005 |
| Coumarins | 4.18 |
| Flavonoids | 0.0 |
| Hydroxycinnamic | 9.26 |
| Other compounds | 25.77 |
### Chart:
| Category | P. heyniae- Water |
|---|---|
| Condensed tannins | 103.76999999999998 |
| Hydrolisable tannins | 3.8200000000000003 |
| Coumarins | 9.1 |
| Flavonoids | 0.0 |
| Hydroxycinnamic | 7.609999999999999 |
| Other compounds | 25.799999999999997 |

## Slide 3
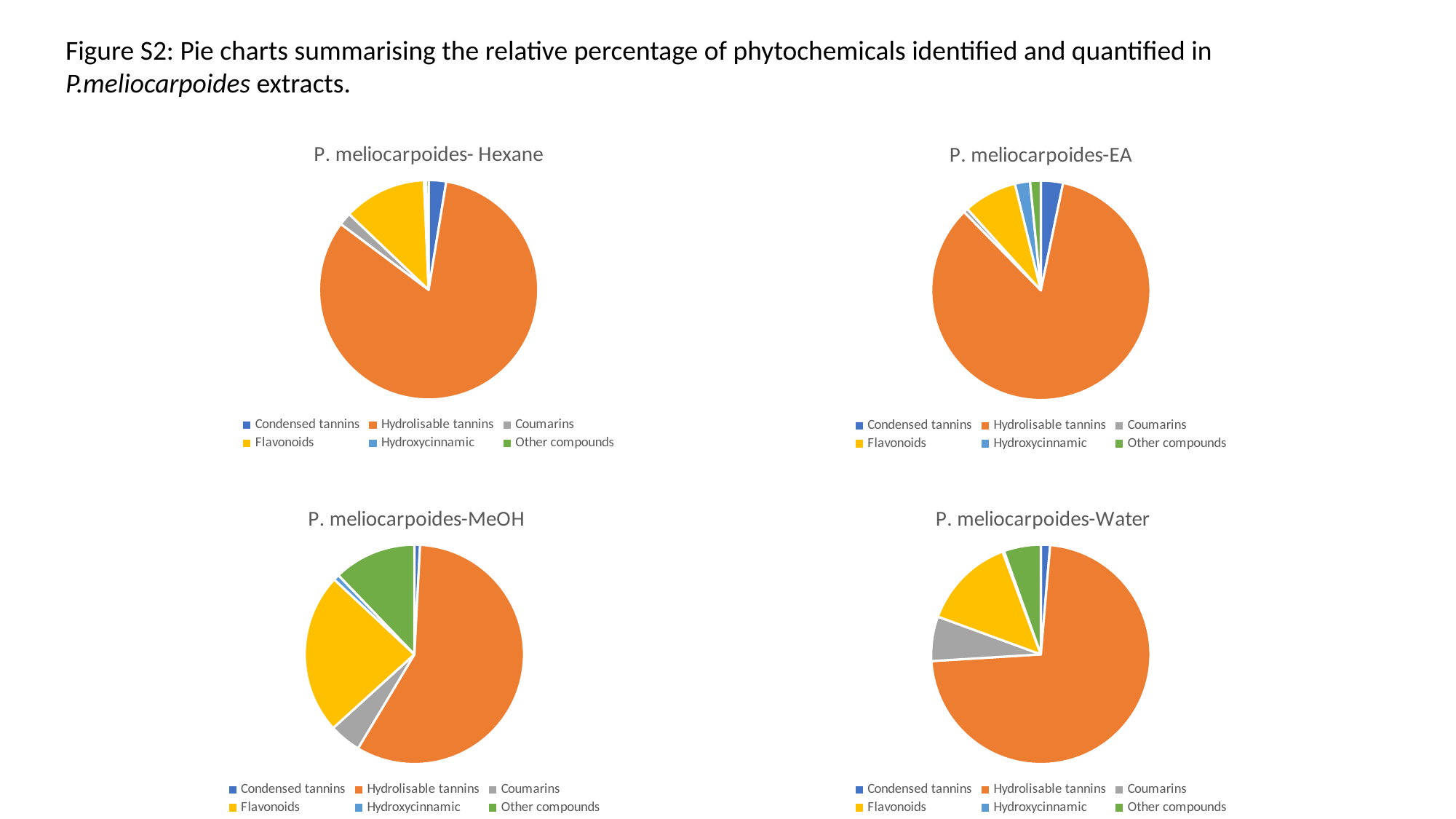

Figure S2: Pie charts summarising the relative percentage of phytochemicals identified and quantified in P.meliocarpoides extracts.
### Chart:
| Category | P. meliocarpoides- Hexane |
|---|---|
| Condensed tannins | 1.29 |
| Hydrolisable tannins | 41.28999999999999 |
| Coumarins | 0.94 |
| Flavonoids | 6.1 |
| Hydroxycinnamic | 0.14 |
| Other compounds | 0.2 |
### Chart:
| Category | P. meliocarpoides-EA |
|---|---|
| Condensed tannins | 2.1500000000000004 |
| Hydrolisable tannins | 55.519999999999996 |
| Coumarins | 0.43000000000000005 |
| Flavonoids | 5.19 |
| Hydroxycinnamic | 1.46 |
| Other compounds | 1.04 |
### Chart:
| Category | P. meliocarpoides-MeOH |
|---|---|
| Condensed tannins | 2.9699999999999998 |
| Hydrolisable tannins | 204.04000000000002 |
| Coumarins | 16.509999999999998 |
| Flavonoids | 83.65 |
| Hydroxycinnamic | 3.07 |
| Other compounds | 43.019999999999996 |
### Chart:
| Category | P. meliocarpoides-Water |
|---|---|
| Condensed tannins | 4.7299999999999995 |
| Hydrolisable tannins | 251.9 |
| Coumarins | 22.82 |
| Flavonoids | 47.62 |
| Hydroxycinnamic | 0.77 |
| Other compounds | 18.919999999999998 |

## Slide 4
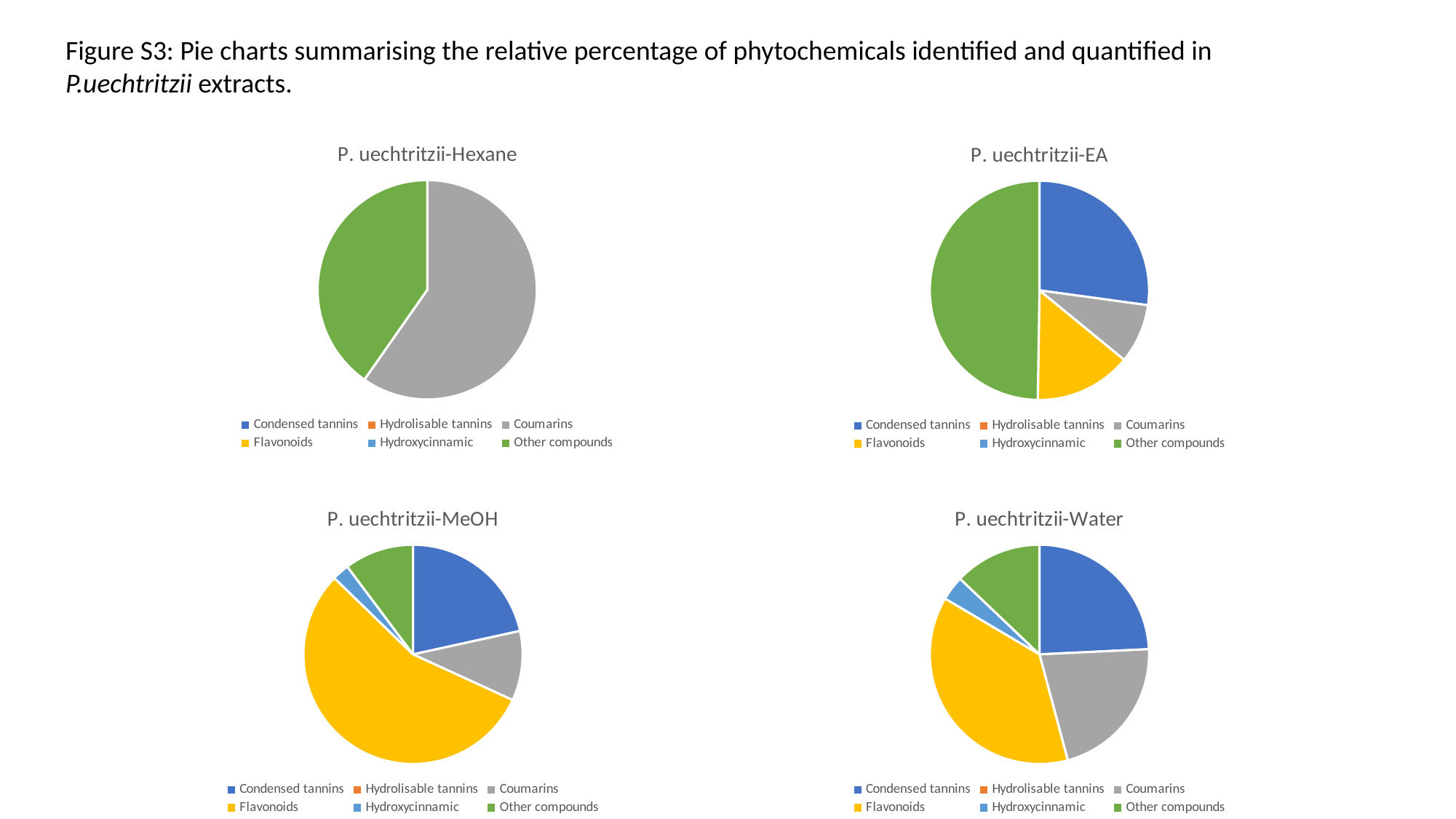

Figure S3: Pie charts summarising the relative percentage of phytochemicals identified and quantified in P.uechtritzii extracts.
### Chart:
| Category | P. uechtritzii-Hexane |
|---|---|
| Condensed tannins | 0.0 |
| Hydrolisable tannins | 0.0 |
| Coumarins | 4.12 |
| Flavonoids | 0.0 |
| Hydroxycinnamic | 0.0 |
| Other compounds | 2.78 |
### Chart:
| Category | P. uechtritzii-EA |
|---|---|
| Condensed tannins | 5.09 |
| Hydrolisable tannins | 0.0 |
| Coumarins | 1.6400000000000001 |
| Flavonoids | 2.68 |
| Hydroxycinnamic | 0.0 |
| Other compounds | 9.32 |
### Chart:
| Category | P. uechtritzii-MeOH |
|---|---|
| Condensed tannins | 53.74 |
| Hydrolisable tannins | 0.0 |
| Coumarins | 25.59 |
| Flavonoids | 138.03 |
| Hydroxycinnamic | 6.27 |
| Other compounds | 25.5 |
### Chart:
| Category | P. uechtritzii-Water |
|---|---|
| Condensed tannins | 31.49 |
| Hydrolisable tannins | 0.0 |
| Coumarins | 28.05 |
| Flavonoids | 48.92 |
| Hydroxycinnamic | 4.69 |
| Other compounds | 16.75 |

## Slide 5
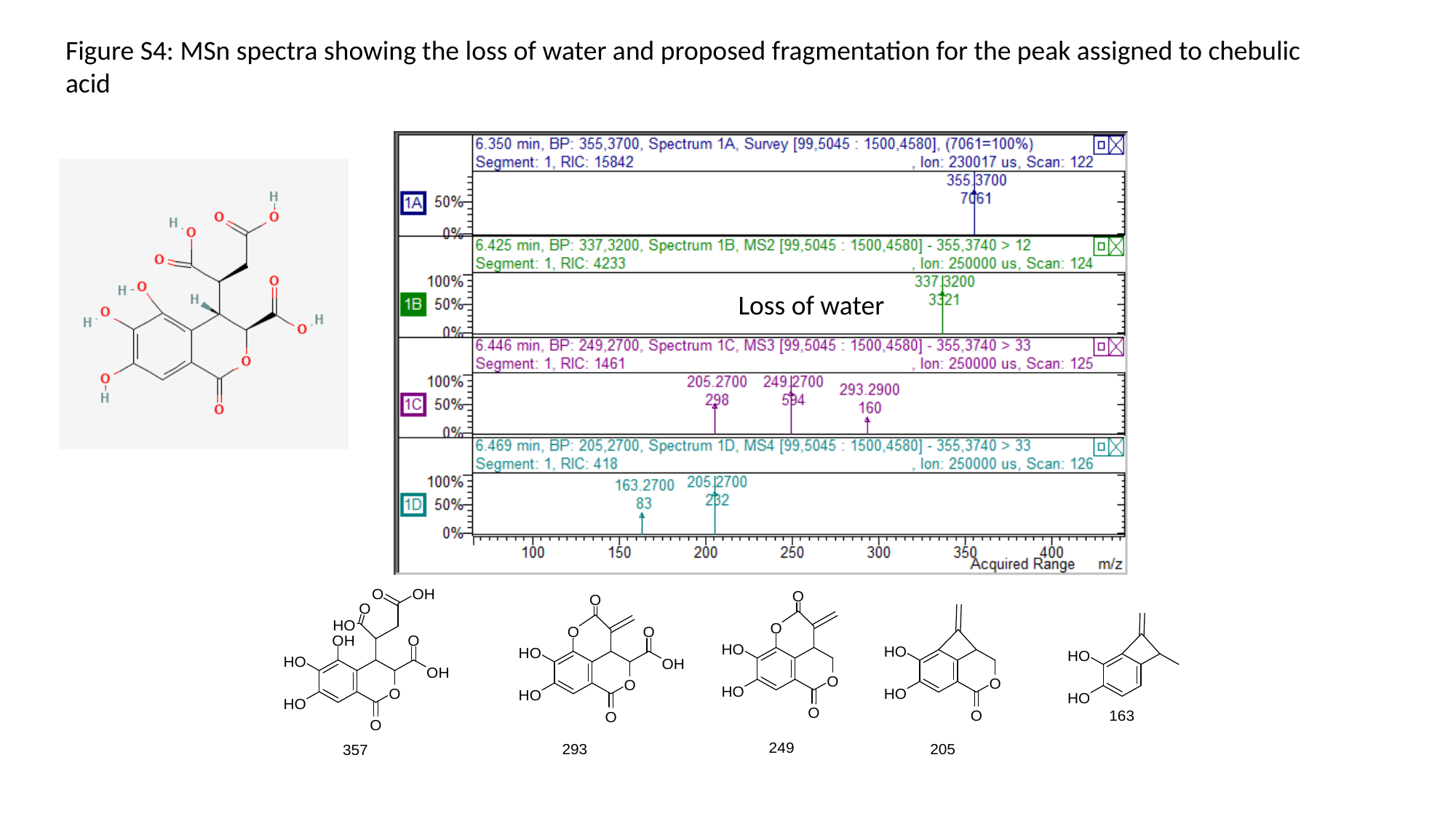

Figure S4: MSn spectra showing the loss of water and proposed fragmentation for the peak assigned to chebulic acid
Loss of water

## Slide 6
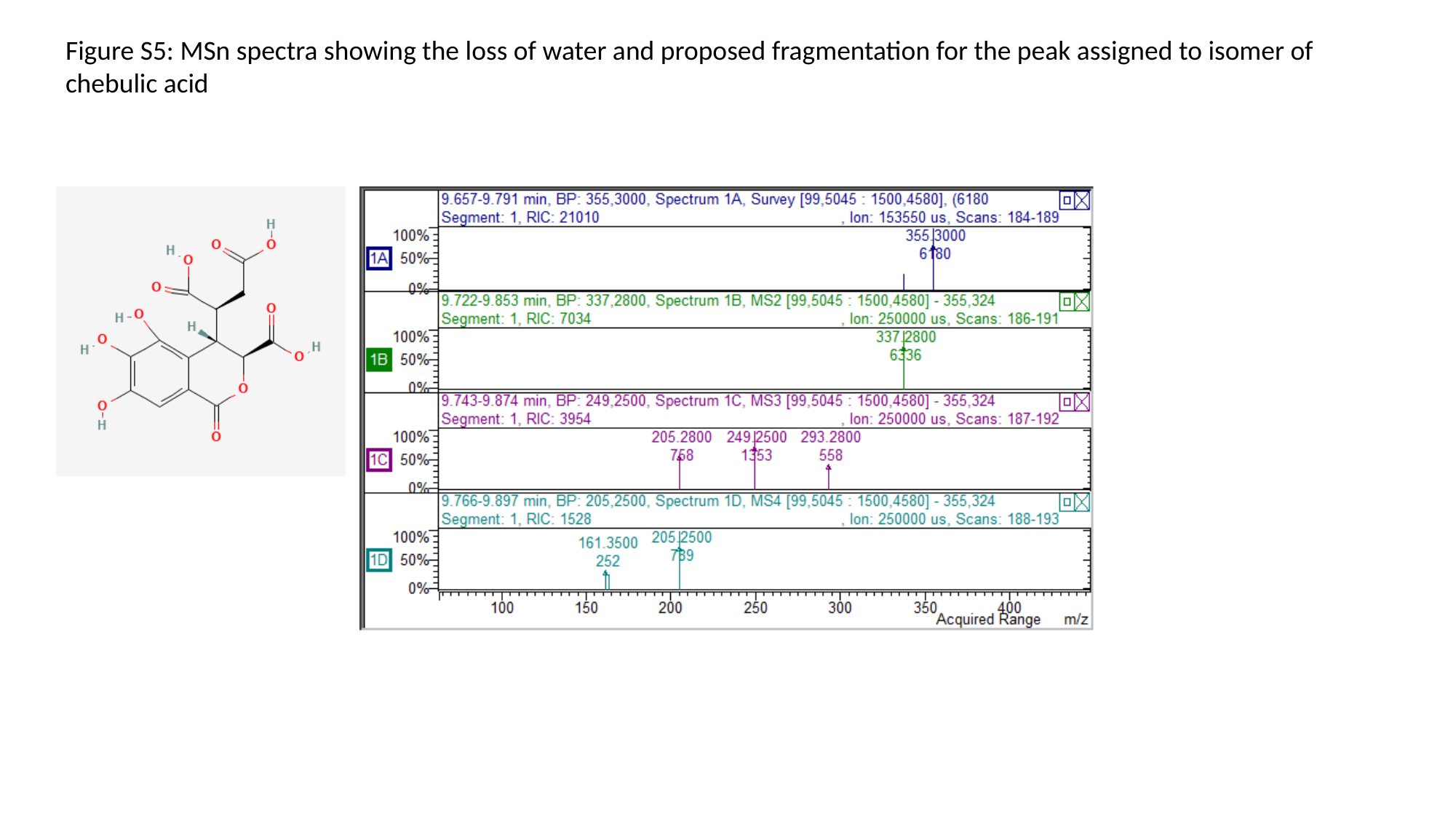

Figure S5: MSn spectra showing the loss of water and proposed fragmentation for the peak assigned to isomer of chebulic acid
